# Characterization and expression profiling of PIN auxin efflux transporters reveal their role in developmental and abiotic stress conditions in rice

**DOI:** 10.3389/fpls.2022.1059559

**Published:** 2022-12-01

**Authors:** Mrinalini Manna, Balakrishnan Rengasamy, Navin Kumar Ambasht, Alok Krishna Sinha

**Affiliations:** ^1^ National Institute of Plant Genome Research, Aruna Asaf Ali Marg, New Delhi, India; ^2^ Department of Botany, Christ Church College, Kanpur, India

**Keywords:** *Oryza sativa* L., canonical and noncanonical PINs, expression patterns, abiotic stress, auxin, root architecture

## Abstract

The auxin efflux transporter proteins called PINs ferry auxin from its source to sinks in particular directions depending on their polar localizations in the plasma membrane, thus facilitating the development of the entire plant architecture. The rice genome has 12 PIN genes distributed over eight chromosomes. To study their roles in plant development, abiotic stress responsiveness, and shaping an auxin-dependent root architecture, a genome-wide analysis was carried out. Based on phylogeny, cellular localization, and hydrophilic loop domain size, the PINs were categorized into canonical and noncanonical PINs. PINs were found expressed in all of the organs of plants that emphasized their indispensable role throughout the plant’s life cycle. We discovered that *PIN5C* and *PIN9* were upregulated during salt and drought stress. We also found that regardless of its cellular level, auxin functioned as a molecular switch to turn on auxin biosynthesis genes. On the contrary, although PIN expression was upregulated upon initial treatment with auxin, prolonged auxin treatment not only led to their downregulation but also led to the development of auxin-dependent altered root formation in rice. Our study paves the way for developing stress-tolerant rice and plants with a desirable root architecture by genetic engineering.

## Introduction

The first discovered phytohormone, auxin, is a vital regulator of plant development because of its ability to provide instructive cues for cell division (Perrot-Rechenmann, 2010), cell elongation (Velasquez et al., 2016), cell differentiation (Marhava et al., 2018), and organogenesis (Braybrook, 2019) in the entire plant. The role of auxin is indispensable throughout the plant’s life cycle starting from early embryogenesis (Robert et al., 2018) to carving out the entire plant architecture (Zhao, 2010). The hormone is involved in the establishment of embryonic apical–basal polarity (Robert et al., 2013), vascular tissue differentiation (Biedroń and Banasiak, 2018), apical hook formation (Béziat and Kleine-Vehn, 2018), apical dominance (Kebrom, 2017), establishment of root architecture (Lavenus et al., 2016), leaf venation patterning (Perico et al., 2021), flowering (Cucinotta et al., 2021), fruit ripening (Cruz et al., 2018), phototropism (Rahman et al., 2010; Rakusová et al., 2011; Leitner et al., 2012; Rakusová et al., 2016), geotropism (Ding et al., 2011; Haga and Sakai, 2012; Zhang et al., 2013), and so on. In order to carry out such diversified activities, it is essential for the hormone to be differentially distributed over the entire plant system from the source of its synthesis. Auxin is typically synthesized in the shoot apical meristem and leaf primordia and transported to targeted plant tissues by bulk flow through the vascular bundle and by unique polar transport machinery (Swarup and Bennett, 2003; Swarup and Péret, 2012). More than one type of auxin transporters participate in the polar auxin transport (PAT), such as PIN-FORMED (PIN), PIN-Like transporters (PILS), ATP-binding cassette (ABC) transporters, AUXIN1/LIKE-AUX1 (AUX/LAX), nitrate transporter 1.1 (NRT1.1), and WALLS ARE THIN 1 (WAT1) (Swarup et al., 2008; Krouk et al., 2010; Barbez et al., 2012; Peret et al., 2012; Swarup and Péret, 2012; Ranocha et al., 2013; Geisler et al., 2017; Barbosa et al., 2018; Ung et al., 2022).

Exclusively found in the plant kingdom, the PIN protein family is an important component of PAT. It belongs to the large bile/arsenite/riboflavin transporter (BART) superfamily, which contains bile, arsenite, and riboflavin transporters distributed across all kingdoms of life (Mansour et al., 2007; Chen et al., 2011). The complete functional PIN protein is constituted by two 5-transmembrane (TM) helix repeats separated by a cytosolic hydrophilic loop (HL) domain that is the site of posttranslational modifications such as phosphorylation that facilitates PIN transportation to the cell membrane and their polar localization (Zourelidou et al., 2014; Zwiewka et al., 2019). The PAT *via* PIN transporters happens as per the well-accepted chemiosmotic model (Leyser, 1999). This model highlights the fact that all PIN proteins studied so far display polar subcellular localization, barring a few that can be found in specific cell types without noticeable polarity. If we assume the cell to be rectangular, polar localization of PIN transporters means that these proteins are localized only in the upper/lower/one of the lateral sides of the cell membrane and not distributed in the entire cell membrane. When inside the cytosol where pH is about 7, auxin (IAA; indole-3-acetic acid; pKa = 4.7) mostly exists in the anionic form (IAA^-^) and becomes trapped inside the cell. PIN auxin efflux carrier proteins have affinity to this deprotonated form of auxin and it facilitates the export of IAA^-^ into the intercellular space. The pH of the intercellular space is acidic (about 5), and here IAA^-^ combines with H^+^ to become an uncharged IAA molecule. The IAA is now received by auxin influx carrier proteins of the adjacent cell and pumped into it. Thus, it continues the unidirectional PAT (Gälweiler et al., 1998; Leyser, 1999; Benková et al., 2003; Vieten et al., 2007). Depending on their polar localization patterns, PINs facilitate sideways, upward, or downward PAT, thus ensuring differential distribution of auxin throughout the plant system that is vital for cellular differentiation and specialized organ formation (Sieberer and Leyser, 2006).

Due to its key role in the differential distribution of auxin inside the plant system, studies involving PIN proteins are important areas of research. The genome of rice (*Oryza sativa* L.), the most important cereal crop of the Asian subcontinent, encodes 12 PIN genes. There have been past genome-wide studies involving rice PIN genes that have illuminated tissue-specific expression of PIN genes and phytohormone-inducible expression of these genes (Wang et al., 2009; Miyashita et al., 2010). However, our genome-wide study highlights different aspects of PIN expression patterns and provides novel insights regarding the involvement of PIN proteins in abiotic stress responsiveness, auxin concentration- and time-dependent PIN expression, regulatory elements present in PIN promoters, co-expressed genes, and most importantly, participation of PINs in carving out root architecture in rice seedlings. The present study provides potential ideas for developing stress-tolerant rice plants and provides new insights related to the response of rice roots to hormonal cues.

## Materials and methods

### Screening PIN genes in the rice genome and analyzing their characteristics

Publicly available genome databases (Rice Genome Annotation Project, http://rice.uga.edu/; NCBI, https://www.ncbi.nlm.nih.gov/; UniProt, https://www.uniprot.org/) were used to find the PIN protein and CDS (coding sequence) and genomic DNA sequences from rice and other plants mentioned in the present study. The molecular weight (MW) and isoelectric point (pI) of PIN proteins were analyzed using Compute pI/Mw tool of Expasy (https://web.expasy.org/compute_pi/). The subcellular protein localizations were predicted using Plant‐mPLoc server (http://www.csbio.sjtu.edu.cn/bioinf/plant-multi/) and web WoLF PSORT tool (https://www.genscript.com/wolf-psort.html). The exon-intron organization of PIN genes was analyzed using the Gene Structure Display Server 2.0 (http://gsds.gao-lab.org/) based on comparison of the CDS sequences with the corresponding genomic DNA sequences. Conserved motifs were found out using the MEME tool (https://meme-suite.org/meme/) with the maximum number of motifs set to 10. The organization of TM helices of PIN proteins in the cell membrane and their three-dimensional (3D) structures were generated using PHYRE2 Protein Fold Recognition Server (http://www.sbg.bio.ic.ac.uk/~phyre2/html/page.cgi?id=index) (**Supplementary Figure S1**). The pore morphology of PIN proteins was predicted using PoreWalker software (https://www.ebi.ac.uk/thornton-srv/software/PoreWalker/) (**Supplementary Figure S1**). The phosphorylation of the HL domain of PIN proteins was predicted by NetPhos 3.1 tool (https://services.healthtech.dtu.dk/service.php?NetPhos-3.1) by using default parameters and MusiteDeep tool (https://www.musite.net/) by setting the cutoff limit at 0.8. The cis‐acting elements present in the promoters (2,000 bp upstream of the start codon) of PIN genes were analyzed using PlantCARE tool (https://bioinformatics.psb.ugent.be/webtools/plantcare/html/), and all of the *cis*-acting elements present in the promoters were tabulated with the help of TBtools v.1098689 software.

### Chromosomal synteny

The chromosomal distribution map for the rice PIN genes was constructed by using CIRCOS Circular Genome Data Visualization software.

### Multiple sequence alignment and phylogenetic analysis

**Figure 1:**
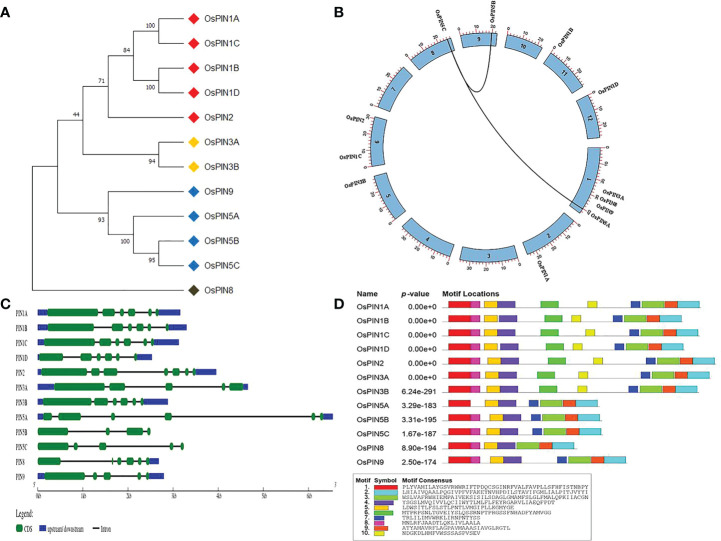
Phylogeny, chromosomal location, gene structure, and protein domains in 12 PINs of rice. **(A)** The phylogenetic tree of PIN proteins showing relative evolutionary relationships among themselves. **(B)** Genome organization of rice PIN genes on eight chromosomes of rice. **(C)** Exon-intron distribution of rice PIN genes. **(D)** Conserved protein domains identified in rice PIN proteins. Out of 10 motifs, seven were common in all of the PIN proteins.

**Figure 2:**
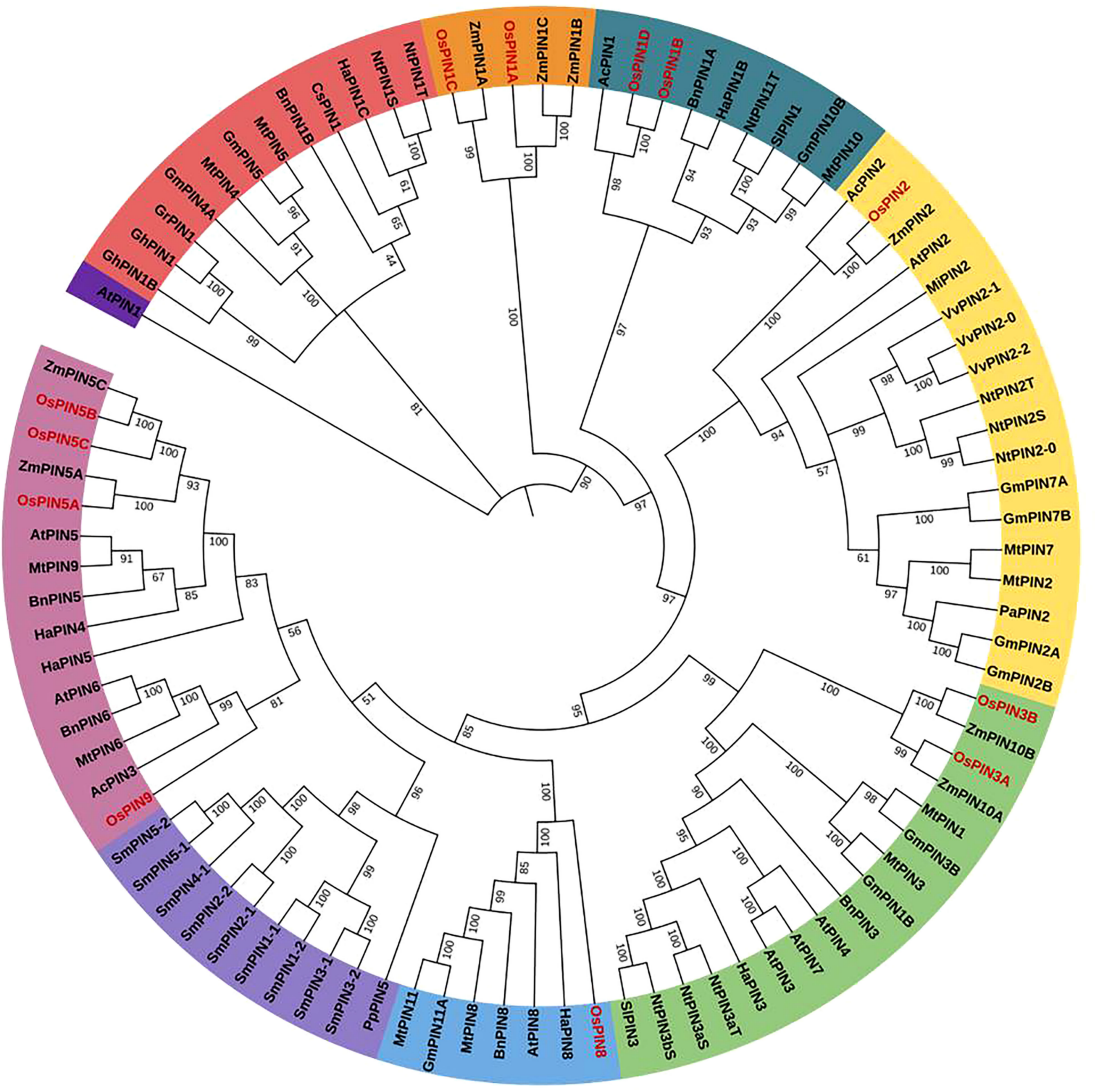
The phylogenetic relationship of PIN proteins of different plant species across the plant kingdom. A maximum likelihood method using 1,000 ultrafast bootstrap replicates was used to construct the tree. The OsPINs are labeled in red. The phylogenetic tree clustered into eight major groups was depicted in different colors.

Multiple sequence alignment of PIN proteins was done using the Clustal Omega program (https://www.ebi.ac.uk/Tools/msa/clustalo/) with default parameters, and the phylogenetic tree (**Figure 1A**) was generated according to the maximum likelihood (ML) method (Truszkowski and Goldman, 2016), and 1,000 replicates were used for bootstrap analysis by using MEGA-11 software. For **Figure 2**, the phylogenetic tree was constructed by IQ-Tree v.2.1.2 (Minh et al., 2020) with 1,000 ultrafast bootstrap replicates using the alignment file generated through MAFFT tool v.7 (Katoh et al., 2019) with default parameters. The tree was visualized and edited using iTOL (v.6) (Letunic and Bork, 2019).

### *In silico* identification of the PIN’s co-expressed and interacting genes

To find out the co-expressed and interacting genes of the rice PINs, STRING software (https://stringdb.org/cgi/input?sessionId=b6H2kGl5OoDW&input_page_active_form=single_sequence) was used with minimum required interaction score set to middle confidence, that is, 0.400 (Szklarczyk et al., 2015). For better visualization of protein–protein interactions, the STRING software-generated network was modified by Cytoscape 3.9.1 software without hampering the interaction pathway and patterns.

### Heat map depicting the expression profile of the PIN genes in different tissues of rice

The rice PIN gene’s expression data (Fragments Per Kilobase of exon per Million mapped fragments or FPKM values) were retrieved from the Rice Expression Database (http://expression.ic4r.org/query?gene=LOC_Os02g50960&cutoff=), and the heat map was generated with the help of HemI 2.0-Heatmap Illustrator software (https://hemi.biocuckoo.org/) using log_2_(FPKM) gene expression values.

### Abiotic stress and IAA/TIBA treatments and expression analysis by qRT-PCR

For subjecting the rice seedlings (var. Taipei 309) to abiotic stress treatments, rice seeds were germinated and grown in half MS (Murashige and Skoog) liquid medium inside a growth chamber at 28°C ± 2°C and 16-h photoperiod conditions for 15 days following which different stress treatments were given. The seedlings growing in the hydroponic solution were transferred inside 42°C incubator (with lights on) to induce heat stress. For salinity stress, the rice seedlings were transferred to half MS solution containing 200 mM sodium chloride (NaCl), and drought stress was given by transferring the rice seedlings to half MS solution containing 260 mM mannitol. Seedlings growing without any stress served as the corresponding control sample. After exposure to 16 h of stress, the whole seedlings were collected, and RNA (ribonucleic acid) was isolated using TRIzol reagent according to the manufacturer’s instructions (Invitrogen).

To analyze the auxin-inducible expression of PIN and auxin biosynthesis genes, 15-day hydroponically grown seedlings (the liquid medium and growth conditions were the same as above) were transferred to fresh liquid medium containing 1 mg/L IAA and grown for 3 h following which the root and leaf tissue samples were harvested for RNA isolation.

For analyzing the auxin-dependent root morphology, rice seeds were grown in half MS solid medium containing increasing concentrations of IAA or TIBA (triiodobenzoic acid). For this, rice seeds were dehusked and surface sterilized with 70% ethanol for 2 min followed by vigorous rinsing with 4% sodium hypochlorite solution and one drop of Tween 20 for 20 min. The seeds were then repeatedly washed with autoclaved sterile water until the smell of chlorine was gone, and after drying, the seeds were placed over the aforesaid IAA/TIBA-containing media for 15 days following which whole seedling and root morphologies were photographed. Morphological parameters were recorded, and shoot and root tissue samples were harvested for RNA isolation using TRIzol reagent. For the combined treatment of IAA and TIBA, the surface sterilized seeds were inoculated over respective IAA+TIBA-containing media and grown for 15 days before being photographed and analyzed.

The quality of the isolated RNA was assessed by gel electrophoresis, and first-strand cDNA was synthesized after DNase treatment according to the manufacturer’s instructions (Thermo Scientific). PCR primers were designed from the 3′ end regions of genes by using the Primer BLAST tool of NCBI (https://www.ncbi.nlm.nih.gov/tools/primer-blast/). qRT-PCR (quantitative real time reverse transcriptase polymerase chain reaction) (as well as semiquantitative RT-PCR for PIN8; **Supplementary Figure S3**) was conducted using the primers (**Supplementary Table S1**) and calculated using rice 18S gene as endogenous control. The experiment was performed in triplicate using three biological replicates for each sample. The relative expression quotient for each PIN gene was calculated using the delta Ct or comparative Ct value method (Livak and Schmittgen, 2001). The fold change was calculated by conversion of the relative expression value (2^-ddCt^) into log_2_ scale. The log_2_(fold change) values above or below 1.5 were considered significant.

### Recording and representing rice root architecture

For photographing root morphology, the entire root was dissected out from the base of the stem and was carefully spread over a solid medium of Phytagel. Roots were photographed (**Supplementary Figure S2**), and for making the exact replica of these root images, root pictures were opened in Adobe Photoshop, and the root outlines were hand drawn over the real root images.

### Gus staining of rice seedlings

Gus (glucuronidase) staining of the rice seedlings was performed by submerging the entire rice seedlings into the solution containing gus buffer (50 mM sodium phosphate buffer, 2 mM potassium ferrocyanide, 2 mM potassium ferricyanide, 1% Triton X) and 1 mg/ml X-gluc (GoldBio Company; Cat. no. G1281C1) overnight at 37°C. To remove the chlorophyll from the leaves, the seedlings were dipped in 100% ethanol and kept overnight at room temperature with shaking. The intensity of gus staining toward the root tip regions was quantified by ImageJ software in which “Histogram” option under the “Analyze” section was used to find the mean intensity values. The lowest mean value was taken to be 100%, and the rest of the percentage values were calculated accordingly.

### Statistical analysis

All experiments were replicated thrice or more as indicated below the corresponding figures. Standard errors of variations (SEVs) of various measurements were calculated. Means were compared between the treatments at the 0.01 probability level using Student’s t-test.

## Results

### Genome-wide identification, phylogenetic analysis, and gene structure analysis of PIN auxin efflux carriers in rice

Based on previous reports and database searches, 12 PIN genes were identified in the whole genome of rice. **Table 1** lists various characteristic parameters of the PIN genes including gene name, locus ID (as per the Rice Genome Annotation Project), genomic DNA length, CDS length, protein length, molecular weight of the proteins, and their pI. Phylogenetic analysis among the 12 PIN genes revealed that PIN1A, PIN1B, PIN1C, PIN1D, and PIN2 are evolutionarily closely related and so are PIN3A and PIN3B. Furthermore, PIN5A, PIN5B, PIN5C, and PIN9 were found to cluster together, and PIN8 was found to belong to a separate clade in the phylogenetic tree (**Figure 1A**). The rice PIN genes are distributed over eight chromosomes of rice, the relative positions of which are depicted in **Figure 1B**. *PIN1A* is located on chromosome 2; *PIN1B* on chromosome 11; *PIN1C* and *PIN2* on chromosome 6; *PIN1D* on chromosome 12; *PIN3A*, *PIN5A*, *PIN8*, and *PIN9* on chromosome 1; *PIN3B* on chromosome 5; *PIN5B* on chromosome 9; and *PIN5C* on chromosome 8. All of the PIN genes contain introns with their numbers varying from 3 (in *PIN5B*) to 6 (in *PIN2*). Due to their varying intron lengths, *PIN5B* was found to be the smallest (2,500 bp) and *PIN5A* was found to be the largest (6,540 bp) gene among the 12 PIN genes of rice. The gene structure analysis in terms of exon-intron distribution revealed that *PIN1A*, *PIN1B*, *PIN1C*, and *PIN1D* are quite similar, containing six exons and five introns (**Figure 1C**). However, the first exon of *PIN1D* is much smaller than that of the other three. The gene structures of the rest of the PIN genes are quite unique in terms of exon-intron positions and their lengths (**Figure 1C**, **Table 1**).

**Table 1:** List of the 12 PIN genes identified in the rice (*Oryza sativa*) genome.

Gene name	Locus ID	Genomic DNA length (bp)	Exon number	CDS length (bp)	Protein length (aa)	MW (kDa)	pI
**OsPIN1A**	LOC_Os02g50960.1 LOC_Os02g50960.2	3,160 3,160	6 5	1,788 1,725	595 574	64.7 62.4	8.94 8.96
**OsPIN1B**	LOC_Os11g04190.1	3,300	6	1,671	554	59.3	8.85
**OsPIN1C**	LOC_Os06g12610.1	3,129	6	1,779	592	64.3	9.05
**OsPIN1D**	LOC_Os12g04000.1	2,532	6	1,173	390	40.8	8.78
**OsPIN2**	LOC_Os06g44970.1	3,957	7	1,893	630	66.6	9.65
**OsPIN3A**	LOC_Os01g45550.1 LOC_Os01g45550.2 LOC_Os01g45550.3	4,660 4,660 4,660	5 6 5	2,013 1,857 1,794	670 618 597	73.1 65.3 62.9	9.55 6.43 6.43
**OsPIN3B**	LOC_Os05g50140.1 LOC_Os05g50140.2	2,886 2,886	7 6	1,776 1,776	591 591	62.9 62.9	9.72 9.72
**OsPIN5A**	LOC_Os01g69070.1	6,540	6	1,092	363	38.6	9.28
**OsPIN5B**	LOC_Os09g32770.1	2,500	4	1,197	398	42.2	7.02
**OsPIN5C**	LOC_Os08g41720.1	3,237	5	1,074	357	37.7	9.42
**OsPIN8**	LOC_Os01g51780.1	2,685	6	936	311	34.1	9.37
**OsPIN9**	LOC_Os01g58860.1	2,797	5	1,017	426	45.6	8.46

CDS, coding sequence; aa, number of amino acids; MW, molecular weight; kDa, kilo dalton; pI, isoelectric point.

To study the evolutionary relationships of rice PIN proteins (depicted with prefix “Os”) with PIN proteins from other plant species such as *Arabidopsis thaliana* (At), *Phaseolus angularis* (Pa), *Solanum lycopersicum* (Sl), *Glycine max* (Gm), *Medicago truncatula* (Mt), *Zea mays* (Zm), *Physcomitrium patens* (Pp), *Nicotiana tabacum* (Nt), *Selaginella moellendorffii* (Sm), *Cucumis sativus* (Cs), *Helianthus annuus* (Ha), *Ananas comosus* (Ac), *Gossypium hirsutum* (Gh), *Boehmeria nivea* (Bn), *Vitis vinifera* (Vv), *Mangifera indica* (Mi), and *Gossypium raimondii* (Gr), a phylogenetic tree was constructed (**Figure 2**). The evolutionary tree was found to be divided into eight major groups.

The clustering patterns reveal that PIN1A and PIN1C belong to one cluster and so do PIN1B and PIN1D. Furthermore, PIN2 and PIN8 belong to separate groups. PIN3B and PIN3A were found to cluster together, and PIN5A–PIN5C along with PIN9 belonged to the same group. The overall phylogenetic data indicate that various classes of PIN proteins in different plant species mostly evolved from their common ancestors that resulted in their class-wise clustering pattern.

### Protein structure, conserved motifs, transmembrane organization, and localization of PIN transporters

The relative arrangements of conserved structural domains in a protein give idea about its function. Therefore, the rice PIN proteins were searched for their conserved structural domains using MEME program. PIN1A, PIN1B, PIN1C, PIN1D, PIN2, PIN3A, and PIN3B were found to have similar motif types and numbers. PIN5B, PIN5C, and PIN9 had similar motif types, but two structural motifs were found to be missing in them as compared to the aforesaid seven PIN proteins. Although PIN5A and PIN8 have the same number of motifs (seven numbers), their types are different. PIN5A has one motif missing toward the N terminal side, while PIN8 has one motif missing in the middle of the protein as compared to PIN5B and PIN5C (**Figure 1D**).

**Figure 3:**
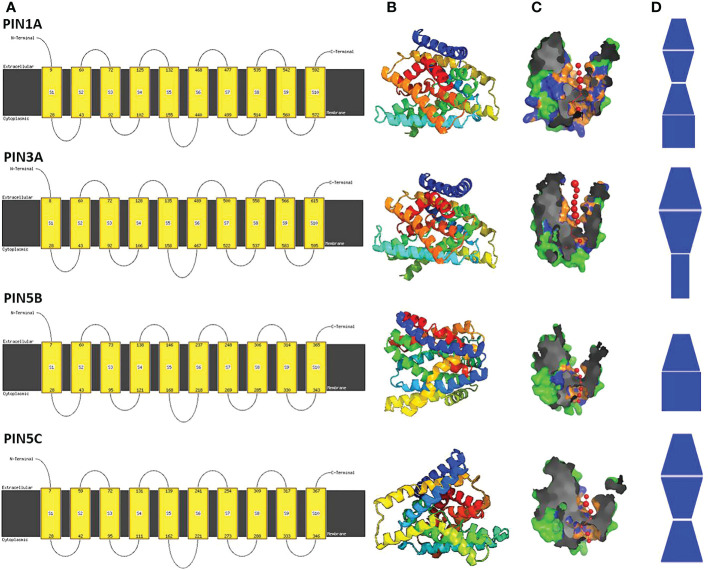
Predicted three-dimensional (3D) models of four rice PIN proteins. **(A)** The cell membrane organization of PIN proteins indicates 10 transmembrane domains and one larger hydrophilic loop (HL) domain present between HL domains 5 and 6. **(B)** The predicted 3D protein structure of PIN proteins indicates the presence of 10 α helices that constitute 10 transmembrane domains. **(C)** Pore morphology of the PIN proteins. **(D)** Pore shapes of PIN proteins indicate varying pore dimensions and morphologies.

The functional PIN proteins are known to contain two 5-TM helix repeats separated by a cytosolic HL domain. Thus, PIN proteins have a total of 10 TM helices with the HL domain present between TM helices 5 and 6. All of the rice PIN proteins were found to have similar TM organization and 3D structure (**Figures 3A, B**; **Supplementary Figure S1**) but differential pore architecture with variation in pore diameter and pore angles (**Figures 3C, D**; **Supplementary Figure S1**). This indicates that PIN proteins might have different affinities toward IAA and different rates of auxin transportation through them.

The HL domains of PIN proteins are important sites for mitogen-activated protein kinase (MAPK)-mediated phosphorylation that determines their polar localization in the cell membrane (Jia et al., 2016). **Table 2** depicts the lengths of the HL domains and the presence of SP/TP (Serine-Proline/Threonine-Proline) motifs in the domain that are the important sites for MAPK-mediated phosphorylation. The HL domains of PIN proteins were found to be of varying lengths ranging from 15 amino acids in PIN1D and PIN8 to 316 amino acids in PIN2 (**Table 2**). Overall, PIN1D, PIN5A, PIN5B, PIN5C, and PIN8 have short HL domains ranging from 15 to 60 amino acids in length, and the rest of the PIN proteins have longer HL domains ranging from 110 to 316 amino acids in length. PIN5C is the only short HL domain containing a PIN that had one SP motif, and it was predicted to be a putative MAPK phosphorylation site. Other short HL domains containing PIN proteins neither had SP/TP motif(s) nor were predicted to be phosphorylated. PINs with long HL domains had variable numbers of SP/TP motifs, and the numbers were proportional to the HL domain’s chain length. All such PIN proteins were predicted to be phosphorylated (**Table 2**).

**Table 2:** Rice PIN protein characteristics (TM, number of transmembrane domains; aa, amino acids; HL, hydrophilic loop).

PIN protein	TM	No. of aa inHL domain (from - to)	SP/TP motifs in HL domain	Phosphorylation(NetPhos, Musite)	Cellular localization (Plant-mPLoc)	Cellular localization (WoLF PSORT)	Tissue-specific PIN expression (based on reports by Wang et al., 2009, and Miyashita et al., 2010)HE: High expression; LE: Low expression
**PIN1A**	10	286 (155 - 440)	SP: 2, TP: 3	Yes, Yes	Cell membrane,	Cell membrane	HE: leaf, stem, stem base, hull vein, anther, young panicle, root, root cap, callus
**PIN1B**	10	246 (158 - 403)	SP: 2, TP: 1	Yes, Yes	Cell membrane	Cell membrane	HE: leaf, stem, stem base, vascular tissues, stele, young panicle, hull vein, stigma, root, adventitious root primordia, root cap LE: shoot apex
**PIN1C**	10	283 (155 - 437)	SP: 2, TP: 4	Yes, Yes	Cytoplasm	Cell membrane	HE: stem, stem base, stele, hull vein, anther, lateral root primordia, root cap LE: leaf, young panicle, root, callus
**PIN1D**	10	15 (154 - 168)	None	No, No	Cell membrane, Nucleus	Endoplasmic reticulum	HE: leaf vascular bundle, root, callus LE: shoot apex
**PIN2**	10	316 (163 - 478)	SP: 4, TP: 4	Yes, Yes	Cell membrane	Cell membrane	HE: shoot apex, leaf, stem base, hull vein, vascular tissues, root LE: young panicle, callus
**PIN3A**	10	310 (158 - 467)	SP: 1, TP: 4	Yes, Yes	Cell membrane, Chloroplast, Nucleus	Cell membrane	HE: shoot apex, leaf, stem, stem base, vascular tissues, callus, young panicle, anther, stigma LE: root, pericycle, stele
**PIN3B**	10	279 (161 - 439)	SP: 0, TP: 3	Yes, Yes	Cell membrane	Cell membrane	HE: leaf, hull vein, anther LE: stem, stem base, pericycle, young panicle, root
**PIN5A**	10	54 (160 - 213)	None	No, No	Cell membrane	Cell membrane	HE: shoot apex, leaf, stem, stem base, vascular tissues, young panicle, anther, adventitious root primordia LE: root
**PIN5B**	10	51 (168 - 218)	None	No, No	Cell membrane	Cell membrane	HE: young panicle, hull vein, anther, vascular tissues LE: shoot apex, leaf, stem, stem base, root callus
**PIN5C**	10	60 (162 - 221)	SP: 1, TP: 0	Yes, Yes	Cell membrane	Cell membrane	HE: shoot apex, leaf, young panicle LE: root
**PIN8**	10	15 (154 - 168)	None	No, No	Cell membrane	Cell membrane, Vacuole, Chloroplast, Mitochondria	HE: shoot apex, young panicle, callus
**PIN9**	10	110 (168 - 277)	SP: 2, TP: 0	Yes, Yes	Cell membrane	Cell membrane	HE: shoot apex, leaf, stem base, vascular tissues, stele, pericycle, root, lateral root primordia LE: leaf, stem, young panicle

Barring a few exceptions, most of the PIN proteins studied so far were found to localize either in the cell membrane (mostly) or in the organellar membrane (rarely) (Křeček et al., 2009). Accordingly, protein localization prediction revealed that all of the rice PIN proteins were located in the cell membrane (**Table 2**). However, PIN1C was predicted to have cytoplasmic localization apart from cell membrane localization, while PIN1D had nuclear and endoplasmic reticulum (ER) localization predictions along with cell membrane localization. Furthermore, PIN3A was found to also have chloroplastic and nuclear localization predictions. In the case of PIN8, apart from cell membrane localization, it was predicted to localize to cell organelles such as vacuole, chloroplast, and mitochondria. Earlier, Wang et al. (2009) and Miyashita et al. (2010) extensively characterized tissue/organ-specific PIN localizations in rice, and we have tabulated that information in **Table 2**.

### *Cis*-acting regulatory elements in promoters of rice PIN genes and co-expressed gene analysis

**Figure 4:**
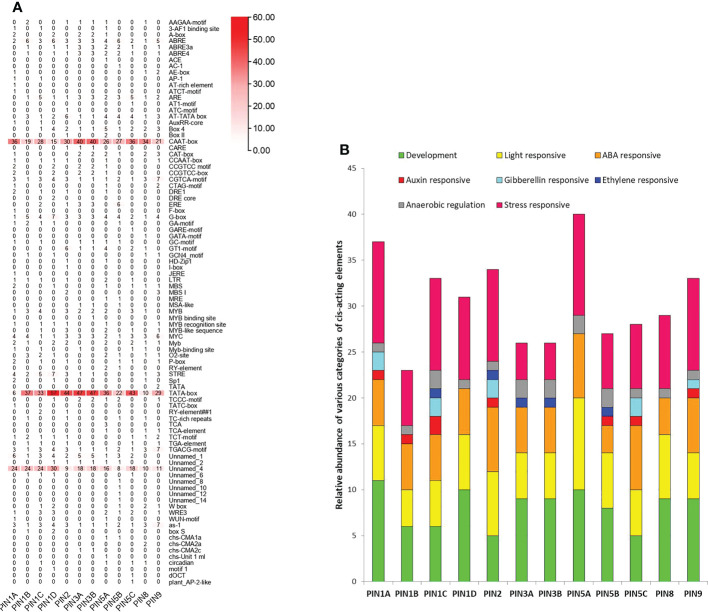
The *cis*-acting regulatory elements present in the 2-kb upstream putative promoter region of rice PIN genes. **(A)** Total number of different *cis*-acting regulatory elements in the respective PIN gene promoters. **(B)** The total number of *cis*-elements was classified into eight categories and their relative abundance in the respective promoters.

A total of 90 *cis*-acting regulatory elements belonging to eight different categories were identified in the promoters of PIN genes (**Figures 4A, B**).

Expectedly, the core regulatory elements, CAAT-box and TATA-box, were the most abundant *cis*-acting regulatory elements present in promoters of all of the PIN genes. A varying number of light regulatory elements (such as 3-AF1 binding site, ACE, AE-box, ATCT-motif, AT1-motif, ATC-motif, Box 4, Box II, G-box, GA-motif, GATA-motif, GT1-motif, I-box, MRE, TCCC-motif, TCT-motif, box S, chs-CMA1a, chs-CMA2a, chs-CMA2c, and chs-Unit 1 ml) were the features of the promoters of all of the PIN genes. Their presence indicates a cross talk between light and auxin signaling in plants. Among other types of *cis*-acting regulatory elements, auxin-responsive (AC-1, AuxRR-core, TGA-element), abscisic acid (ABA)-responsive (ABRE; MYB, MYB binding site, MYB recognition site, MYB-like sequence), stress-responsive (AP1, CCAAT-box, CGTCA-motif, DRE1, DRE core, LTR, MBS, MBSI, STRE, TC-rich repeats, TCA, TCA-element, TGACG-motif, W box, WRE3, WUN-motif, as-1, plant_AP-2-like), and overall plant development-specific (A-box, AT-rich element, AT-TATA box, CAAT-box, CAT-box, CCGTCC motif, CCGTCC-box, CTAG-motif, F-box, HD-Zip 1, GCN4_motif, MSA-like, MYC, O2-site, RY-element, Sp1, TATA-box, circadian, motif 1) regulatory elements were present in more abundance in comparison to gibberellin-responsive (GARE-motif, P-box, TATC-box, RY-element) and ethylene-responsive (ERE) elements in the promoters of PIN genes. However, the promoters of *PIN1A*, *PIN5C*, and *PIN9* lack ERE elements, the *PIN1B* promoter lacks gibberellin- and ethylene-responsive elements, and the *PIN1D* and *PIN8* promoters do not have auxin-, gibberellin-, and ethylene-responsive elements. Furthermore, the promoters of *PIN3A*, *PIN3B*, and *PIN5A* lack auxin- and gibberellin-responsive elements, and the *PIN5B* promoter does not have gibberellin-responsive elements. Rice is grown in puddled soil, and often, it completes a major portion of its life cycle in standing water. Thus, rice roots are adapted for anaerobic respiration. *Cis*-acting regulatory elements specific to anaerobic regulation (such as ARE and GC-motif) that were found in promoters of all of the PIN genes indicating flooding and water scarcity might have a bearing on PIN expression and associated modification in root phenotype and physiology. The overall observation indicates that the expression of rice PIN genes is regulated by light-, stress-, phytohormone-, and development stage-specific signals.

**Figure 5:**
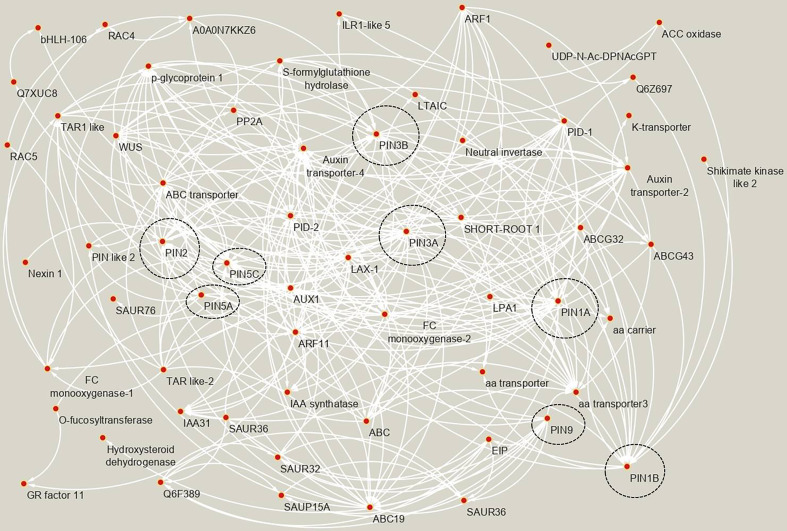
The co-expressed and interacting genes of rice PIN genes as evident by STRING analysis. Each arrow originates at the “source node” and ends at the “target node.” The “source node” corresponds to the “effector protein,” and the “target node” corresponds to the “target protein.” The nodes containing rice PIN proteins are encircled by black borders. All of the proteins mentioned in the network belong to *Oryza sativa*. The full name of the proteins, UniProt IDs, putative functions, and their classification into 12 categories are mentioned in **Supplementary Table S2**.

To find the PIN’s co-expressed and interacting genes in rice, the STRING database was searched, and various categories of genes were found to interact and co-express with the PIN genes (**Figure 5**, **Supplementary Table S2**). The data revealed that various categories of IAA transporters (such as PINs, ABC transporters, AUX1, LAX1), PIN localization regulators (PIDs/protein kinase PINOIDs), IAA biosynthesis- and metabolism-related genes (such as FC-monooxygenase, TAR, and Nexin 1), negative regulators of auxin responsiveness (such as IAA31, SAUR32, SAUR36, and SAUR76), and positive regulators of auxin responsiveness (ARF proteins) are co-regulated and interact among themselves to bring about auxin homeostasis in plants. Additionally, PIN proteins were found to cross talk with other phytohormone signaling-associated proteins. For instance, an ethylene-insensitive class of transcription factors, EIP, was identified to be a target of PIN9 and an ethylene biosynthesis pathway-associated protein, ACC oxidase, was a target of PIN1B protein. Furthermore, GR factor 11 (a transcription factor associated with gibberellin-mediated stem elongation) was found to be an indirect target of multiple PIN proteins *via* auxin response factor ARF11. Amino acid and potassium transporters were also found to co-express and interact with rice PINs. A number of proteins belonging to the “overall plant growth and development” category (such as bHLH-106, influences grain length and weight; LPA1, regulates phytic acid accumulation in seeds; LTAIC, regulates optimum tiller angle; SHORT-ROOT 1, mediates asymmetric cell division in roots) were also found to co-express and interact with rice PIN proteins. Interestingly, we found a Phosphatase 2A regulatory A subunit protein (PP2A; known to provide enhanced resistance to sheath blight disease in rice) having a possible interaction/co-expression with OsPIN2 (**Figure 5**).

### Tissue-specific transcript profiling of PIN genes

**Figure 6:**
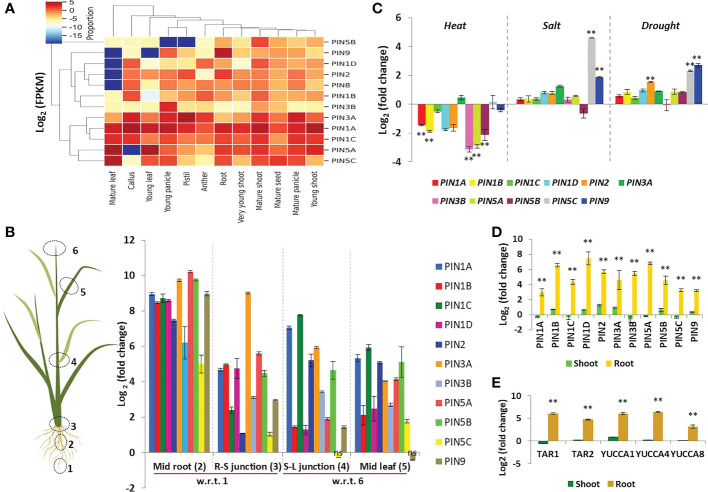
Expression patterns of rice PIN genes in different tissues under different conditions. **(A)** Expression profiling of PIN genes of rice at different developmental stages using RNA sequencing data available at the Rice Expression Database. The color bar in the right-hand side of the heat map represents relative expression values. **(B)** Expression profiling of rice PIN genes in growing (i.e., mid root, 2; mid leaf, 5) and differentiating (i.e., root-shoot or R-S junction, 3; stem-leaf or S-L junction, 4) regions of rice seedlings in comparison to the root tip, 1 region, and leaf tip, 6 region, by qRT-PCR (n = 3, non-significant changes are marked as “ns”). **(C)** Changes in the expression levels of PIN genes in response to heat, salt, and drought stress (n = 3). **(D)** Upregulation of PIN genes in the roots of rice seedlings when subjected to IAA treatment for 3 h (n = 3). **(E)** Induction in the expression of IAA biosynthesis genes in the roots of rice seedlings post 3 h of IAA treatment (n = 3). Asterisks (**) indicate differences statistically significant at 0.01 probability.

Many earlier studies have revealed tissue-specific expression patterns of rice PIN genes, and the Rice Expression Database maintains such data. Therefore, we accessed the database and retrieved the PIN gene expression data to construct a heatmap revealing PIN expression patterns in very young shoot, young root, mature shoot, root, young leaf, mature leaf, anther, pistil, young panicle, mature panicle, mature seed, and callus tissues (**Figure 6A**). Among all of the rice PIN genes, *PIN1A* had a fairly high expression in all of the tissues. *PIN1C*, *PIN3A*, *PIN5B*, and *PIN5C* had moderate levels of expression in all of the aforesaid tissues. The transcript abundance of *PIN5A* was higher in vegetative tissues than that in reproductive tissues. *PIN9* had the highest expression in root as compared to other tissues. *PIN1B*, *PIN1D*, *PIN2*, *PIN3B*, and *PIN8* were found to have a low transcript abundance in all of the tissues.

While documenting the auxin gradient in DR5-gus rice seedlings (to be discussed later in subsequent sections), we found that root and leaf tips were the first to stain blue, depicting the highest concentrations of auxin present in these regions. Therefore, we wanted to investigate the correlation between the auxin level and PIN expression. Furthermore, we wanted to reveal the level of PIN expression in differentiating regions of rice seedlings such as stem-root junction and stem-leaf junctions. The qRT-PCR analysis revealed that the transcript abundance of all of the PINs was the lowest in the leaf tip and root tip regions where auxin concentration was the highest. The growing regions of the rice seedlings (i.e., elongating root region present above the root tips, mid leaf region) and differentiating zones (i.e., leaf-stem junctions, shoot-root junctions) have comparatively more transcript abundance of the PINs. For instance, the root elongation zone (or mid root) and root-shoot junction had a higher expression of all of the PIN genes as compared to their expression in the root tip region (**Figure 6B**). Furthermore, the PIN expression in the stem-leaf junction and mid leaf zones was comparatively higher in relation to its expression in the leaf tip regions (**Figure 6B**). However, *PIN5C* (in stem-leaf junction) and *PIN9* (in mid leaf) had no significant change in the expression level in comparison to their expression in the leaf tips (**Figure 6B**). The overall observations indicated that the PIN proteins play a significant role in auxin distribution in growing and organ-differentiating regions of rice, and their involvement is limited in quiescent zones like the root tips and leaf tips. It is important to note that in our study, the expression of the *PIN8* gene was never detected. Earlier, Miyashita et al. (2010) showed that the expression of rice *PIN8* was confined to the shoot apex (from 6-week-old plants), panicle, and callus and not in the leaf and root tissues (from the same 6-week-old plants). In line with this, we rechecked the expression of *PIN8* in 11 tissue/treatment samples (**Supplementary Figure S3**) and witnessed a weak expression of *PIN8* only in the upper half region of the young panicle. These observations indicate that *PIN8* might have either no expression or very little expression in young shoot and root tissues of rice seedlings.

### Response of PIN genes to abiotic stress treatments

The major abiotic stresses witnessed by a field-growing rice plant are heat, salt, and drought stresses. Auxin signaling is known to participate in abiotic stress tolerance apart from its involvement in plant growth and development. Therefore, we checked the expression pattern of PIN genes after subjecting the rice seedlings to the aforesaid stress conditions. Heat stress resulted in considerable downregulation of *PIN1A*, *PIN1B*, *PIN3B*, *PIN5A*, and *PIN5B* transcript abundance, whereas the transcript level of the rest of the PINs remained unchanged (**Figure 6C**). Salt stress was found to upregulate the expression of *PIN5C* and *PIN9*, indicating their possible involvement in salt stress tolerance in rice (**Figure 6C**). Furthermore, drought stress significantly upregulated *PIN2*, *PIN5C*, and *PIN9*, revealing their possible involvement in regulating auxin flow for drought stress tolerance in rice seedlings (**Figure 6C**).

### A brief period of IAA treatment induces the expression of PINs and IAA biosynthesis genes

Since PIN proteins distribute auxin throughout the plant system, we wanted to investigate if the induction of auxin biosynthesis and the PIN genes are co-regulated. We discovered that 3 h of IAA treatment induced the expression of both IAA biosynthesis genes (tryptophan aminotransferases, *TAR1*, *TAR2*; flavin-monooxygenase-like enzymes, *YUCCA1*, *YUCCA4*, *YUCCA8*) and PIN genes in rice roots (**Figures 6D, E**). This indicates that the increase in auxin biosynthesis triggers the transcription of PIN genes by enabling auxin translocation from the source of its synthesis. It is important to note that the expression of all of these genes was significantly upregulated in root tissues as compared to shoot tissues. The experiment revealed the PIN transporter’s dynamic influence on root development of the rice seedlings.

### Prolonged IAA and TIBA treatment alters the root architecture in rice seedlings

**Figure 7:**
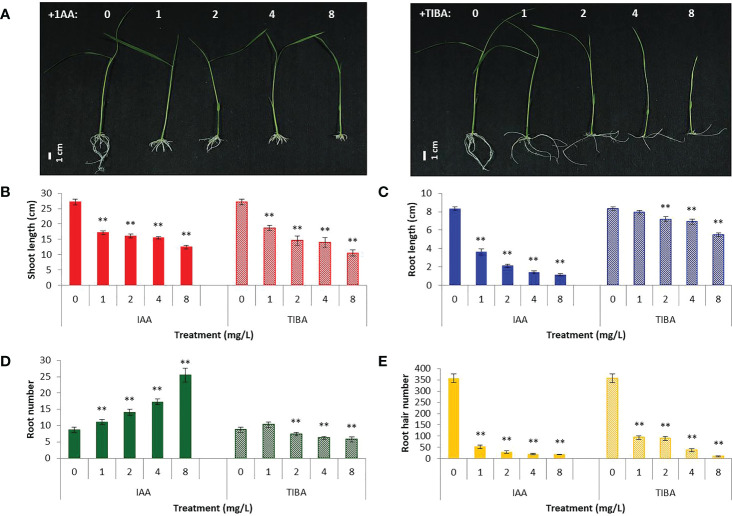
Rice seedling morphology and growth parameters when subjected to increasing levels of IAA and TIBA treatments (n = 10). **(A)** Seedling morphologies when rice seeds were grown over half MS medium containing IAA or TIBA (at 1, 2, 4, and 8 mg/L concentrations) for 15 days. The contrasting root morphologies upon IAA and TIBA treatments are vividly evident. **(B)** Quantification of shoot lengths upon IAA or TIBA treatments. Both treatments reduced the shoot lengths. **(C)** Measurement of the root lengths upon IAA or TIBA treatments. Both treatments reduced the root lengths. **(D)** Quantification of root numbers upon IAA or TIBA treatments. While IAA treatments increase the root numbers, TIBA treatments decrease their numbers. **(E)** Root hair numbers in IAA- and TIBA-treated rice seedlings. Both treatments led to a drastic reduction in root hair numbers. Asterisks (**) indicate differences statistically significant at 0.01 probability.

**Figure 8:**
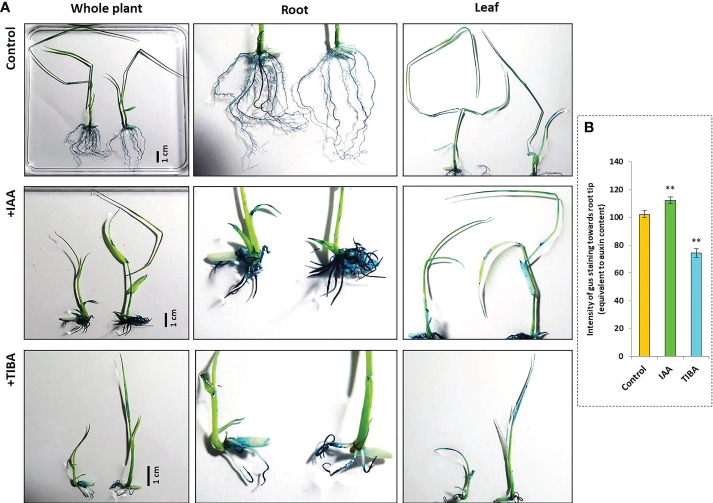
Gus staining of rice seedlings harboring *gus* gene under the control of the auxin-responsive DR5 promoter. **(A)** The rice seeds were germinated and grown over half MS medium containing 4 mg/L IAA or 8 mg/L TIBA for a duration of 15 days following which gus staining was carried out. The intensity of gus stain depicts the level of auxin present inside the tissue. IAA-treated seedling roots stained the most. In TIBA-treated seedlings, the upper part of the roots was not stained, showing the disruption of auxin transportation from the root tip (the zone where auxin accumulates in the highest amount). **(B)** The intensity of gus staining in and around the root tip region under control, +IAA, and +TIBA conditions depicting the level of internal auxin in the root tissues. Asterisks (**) indicate differences statistically significant at 0.01 probability.

**Figure 9:**
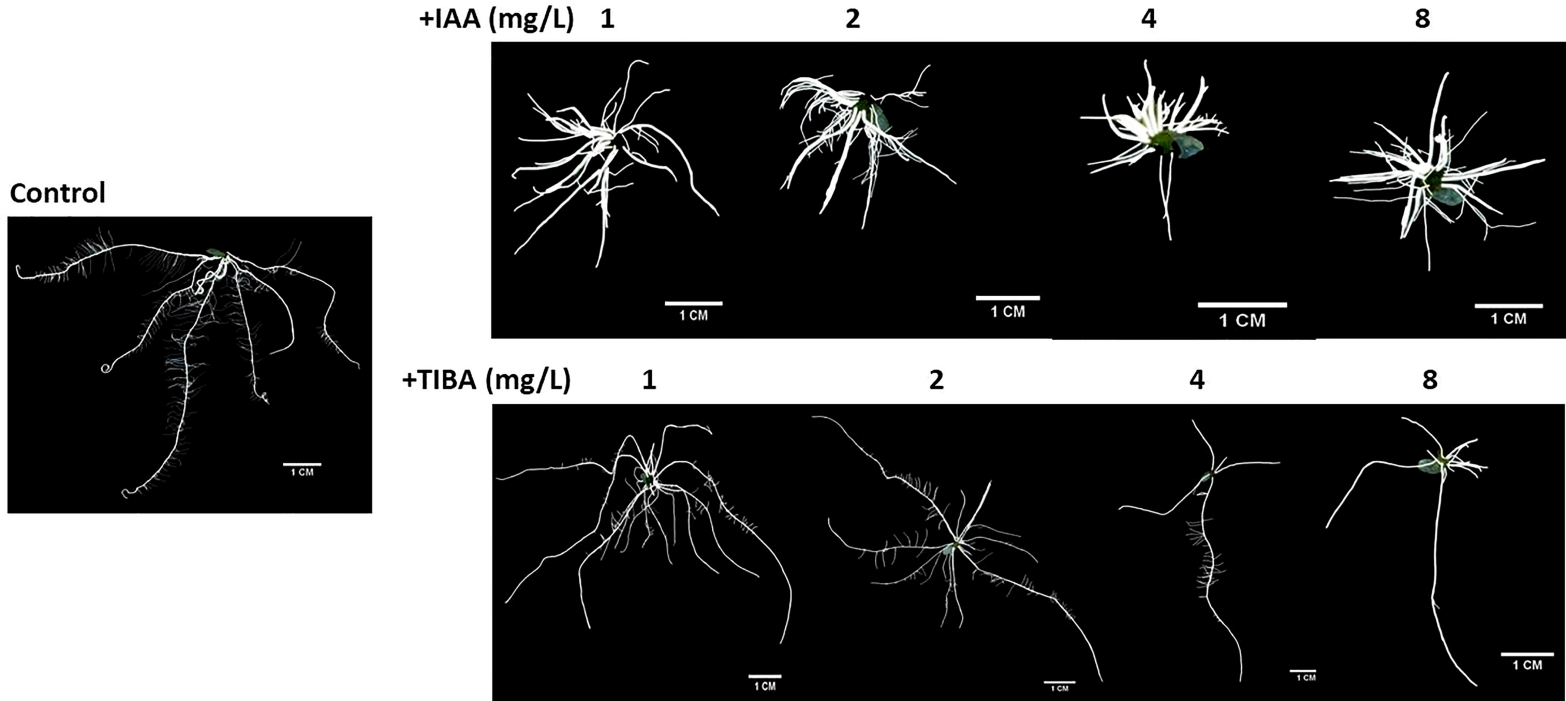
Magnified root images under control and IAA/TIBA treatment conditions.

IAA promotes rooting, and TIBA is an auxin transport inhibitor. We wanted to investigate the response of rice seedlings especially the rice root system to exposure to IAA and TIBA. Therefore, rice seeds were germinated and grown over solid media containing increasing levels of IAA or TIBA (1, 2, 4, and 8 mg/L; equivalent to 5.7, 11.4, 22.8, and 45.6 μM) for 15 days to analyze the plant and root architecture. We observed that both IAA and TIBA treatments led to a gradual reduction of the shoot length as the exposure levels to IAA/TIBA were increased (**Figures 7A, B**; **Supplementary Table S3**). However, IAA treatments had a drastic influence on diminishing rice root lengths. The higher the concentration of auxin, the lower was the root length. Increasing TIBA treatment also led to a decrease in root lengths, but the effect was much milder (**Figure 7C**, **Supplementary Table S3**). Interestingly, we found that increasing IAA treatments led to an increase in the number of secondary root formation in rice seedlings (**Figure 7D**, **Supplementary Table S3**). Furthermore, in order to observe if the IAA-treated rice seedling has really internalized the medium IAA, transgenic rice seeds expressing the *gus* gene under the regulation of the auxin-inducible DR5 promoter were given similar IAA treatments. Deep gus staining of rice roots as compared to the control rice seedlings revealed that the rice roots really had higher levels of endogenous auxin (**Figures 8A, B**). Therefore, it indicates that a high endogenous auxin promotes root formation in rice. However, when auxin flow in the plant was restricted by exposing the rice seedlings to increasing levels of TIBA, the number of roots developed was found to gradually decrease beyond 1 mg/L TIBA treatment (**Figure 7D**). Gus staining of TIBA-treated DR5-gus rice seedlings revealed an interruption in auxin transportation from root tip to root-shoot junction (**Figure 8A**), and TIBA-grown roots had a lower auxin content (**Figure 8B**). Thus, inhibition of auxin transportation was found to affect the root formation in rice. Furthermore, both IAA and TIBA treatments were found to drastically reduce the root hair formation (**Figure 7E**, **Supplementary Table S3**). The detailed images of the root architecture from IAA- and TIBA-treated rice seedlings are shown in **Figure 9**.

What happens when rice seeds are germinated in a medium containing both auxin and an auxin transport inhibitor? We looked into it and found that IAA had more influence over TIBA in shaping the root architecture in rice seedlings. Germinating rice seeds over media containing 1 mg/L IAA + 2 mg/L TIBA or 4 mg/L IAA + 8 mg/L TIBA led to the production of shorter but multiple secondary roots just like as it happens when seeds are germinated over a medium containing only IAA (**Supplementary Figure S4**). However, there was a significant reduction in the root numbers in 4 mg/L IAA + 8 mg/L TIBA treatment in comparison to 4 mg/L IAA treatment (**Supplementary Table S3**). This indicated that while IAA promotes root formation, TIBA acts against it.

### Prolonged IAA treatment downregulates the expression of PIN genes but upregulates the expression of IAA biosynthesis genes

**Figure 10:**
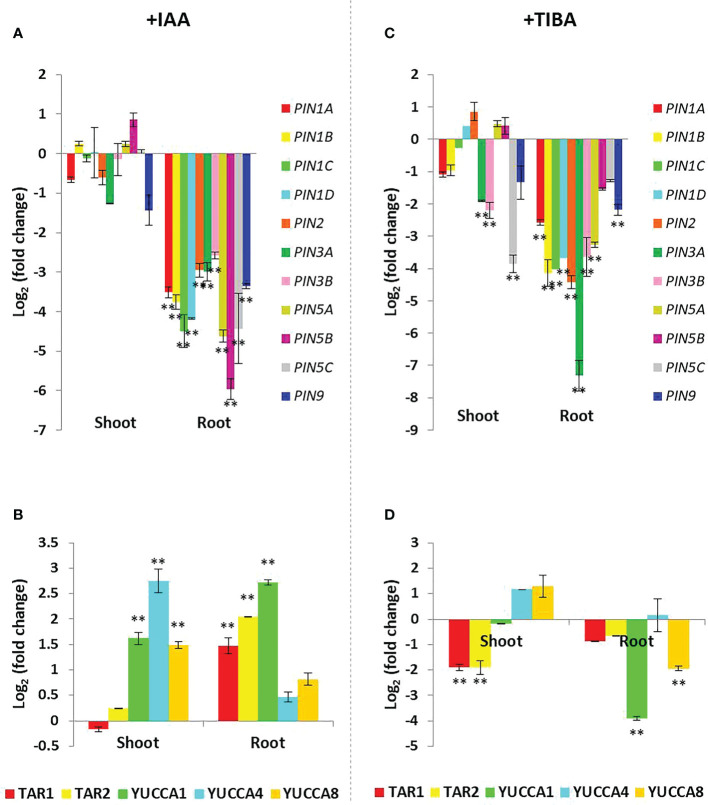
Expression profiling of the PIN and IAA biosynthesis genes in the shoot and root tissues of rice seedlings grown over half MS media containing 4 mg/L IAA or 8 mg/L TIBA for a duration of 15 days. **(A)** Downregulation of the PIN genes in IAA-treated rice seedlings (n = 3) as measured by qRT-PCR. The downregulation is more prominent in the root tissues as compared to the shoot tissues. **(B)** Upregulation of the IAA biosynthesis genes in IAA-treated rice seedlings (n = 3). **(C)** Downregulation of the PIN genes in TIBA-treated rice seedlings (n = 3) as measured by qRT-PCR. The downregulation is more prominent in the root tissues as compared to the shoot tissues. **(D)** Downregulation of the IAA biosynthesis genes in TIBA-treated rice seedlings (n = 3). Significant changes are marked with asterisks (**).

Such a differential influence of IAA and TIBA on rice root development prompted us to check the expression patterns of PIN genes and IAA biosynthesis genes in rice. Unexpectedly, we found that prolonged IAA treatment led to the downregulation of PIN genes, and the extent of PIN downregulation was more pronounced and significant in the root tissues as compared to the shoot tissues (**Figure 10A**). On the contrary, most of the IAA biosynthesis genes were significantly upregulated in both root and shoot tissues (**Figure 10B**). Thus, it indicates that although external auxin stimuli trigger auxin biosynthesis in roots, they lead to the inhibition of auxin transportation by downregulating the expression of PIN genes.

Combined IAA and TIBA treatments were found to downregulate the majority of the PIN genes in rice roots (except PIN9 that was found to be upregulated). Consequently, majority of the IAA biosynthesis genes were upregulated in both the roots and shoot tissues (**Supplementary Figure S4**), a phenomenon associated with a high cellular auxin.

### Prolonged TIBA treatment downregulates the expression of both PIN and IAA biosynthesis genes

When we examined the expression patterns of PIN and IAA biosynthesis genes in TIBA-treated rice seedlings, we observed that TIBA treatment caused an IAA-like PIN gene downregulation, which was more noticeable and substantial in the root tissues than the shoot tissues (**Figure 10C**). However, unlike IAA treatment, TIBA exposure led to the downregulation of auxin biosynthesis genes in the root tissue (**Figure 10D**). In the shoot, *TAR1* and *TAR2* transcripts were significantly downregulated, while *YUCCA1* and *YUCCA8* were significantly downregulated in the root. Therefore, TIBA treatment was found to downregulate the expression of both PIN and IAA biosynthesis genes.

## Discussion

### PINs have the general characteristics of auxin efflux transporter proteins and are conserved during evolution

PINs belong to a class of TM proteins, and their polar localization in the cell membrane facilitates directional flow of auxin in the plant system (Langowski et al., 2016; Nodzyński et al., 2016). The classification of PIN proteins is generally based on phylogenetic relationships, subcellular localization, and most importantly length of HL domains. Accordingly, the PIN protein family members can be grouped into three categories: 1) the canonical PINs [plasma membrane (PM)-localized PINs with a long central HL], 2) the noncanonical PINs [PINs with a very short HL domain that are mostly ER-localized], and 3) the “other category” PINs (dual PM- and ER-localized PINs) (Ganguly et al., 2012; Bennett et al., 2014; Verna et al., 2015; Simon et al., 2016; Glanc et al., 2018). The *Arabidopsis* genome encodes eight PIN genes, and they fall into these three categories: AtPIN1, AtPIN2, AtPIN3, AtPIN4, and AtPIN7 are the canonical PINs with a long central HL domain (>~300 amino acids); AtPIN5 and AtPIN8 belong to the noncanonical PIN category with a short (<~50 amino acids) central HL domain with subcellular localization; and AtPIN6 belongs to the “other category” with a long HL domain but localization in both PM and ER (Ganguly et al., 2014; Yang et al., 2019; Zhang et al., 2020a). Our study revealed that rice PIN1A, PIN1B, PIN1C, and PIN1D had a close evolutionary relationship with AtPIN1. The PIN2 clustered with AtPIN2 while PIN3A and PIN3B clustered with AtPIN3, AtPIN4, and AtPIN7. Furthermore, out of these seven rice proteins (i.e., PIN1A, PIN1B, PIN1C, PIN1D, PIN2, PIN3A, and PIN3B), six (i.e., other than PIN1D) were found to have long HL domains (246–316 amino acids in length) having potential MAPK phosphorylation motifs. These proteins were also predicted to be localized in the cell membrane. Based on these observations, we propose that the six rice PIN proteins, namely, PIN1A, PIN1B, PIN1C, PIN2, PIN3A, and PIN3B are the canonical PINs with long HL domains and possible cell membrane localization. Among other rice PIN proteins, PIN5A, PIN5B, PIN5C, and PIN9 clustered with AtPIN5 and AtPIN6, while rice PIN8 clustered with AtPIN8. In rice, PIN1D, PIN5A, PIN5B, PIN5C, and PIN8 were found to have very short HL domains (15–60 amino acids in length) with no MAPK phosphorylation motifs (except PIN5C), and PIN1D and PIN8 were predicted to have subcellular localizations. Thus, we believe that PIN1D, PIN5A, PIN5B, PIN5C, and PIN8 can be classified as noncanonical PINs with short HL domains. Since PIN9 has a fairly long HL domain (110 amino acids in length) with probable PTM (post-translational modification) sites, localizes in the PM, and clusters with PM-localized AtPIN6, we believe that it is a canonical PIN in rice. Thus, in a nutshell, rice PIN1A–PIN1C, PIN2, PIN3A, PIN3B, and PIN9 have a very high possibility to belong to canonical PINs, and the rest of the five PIN members (PIN1D, PIN5A–PIN5C, and PIN8) are the noncanonical PINs.

Dory et al. (2018) showed that the PIN proteins possess three highly conserved putative MAPK (mitogen-activated protein kinase) sites adjacent to the phosphorylation sites of the well-characterized PINOID kinase proteins that regulate the polar localization of PINs. Moreover, in *Arabidopsis*, MPK6 was shown to phosphorylate S337 motifs present in the HL domain of AtPIN1 and was found to influence polar localization of AtPIN1 in the cell membrane (Jia et al., 2016). Thus, MAPKs are involved in the regulation of PIN auxin efflux transporters. In the present study, canonical rice PINs were found to possess one or more potential MAPK phosphorylation motifs, and therefore, we believe that rice MPKs might have a crucial role in regulating the PIN protein’s activation and localization in the cell membrane. All of the rice PIN proteins regardless of their HL domain characteristics and putative localizations were found to have highly conserved TM domains (**Supplementary Figure S1**), a feature of PIN proteins. The predicted pore morphology of the PIN proteins revealed five different types of pore architecture, and the pore architecture might determine the affinity toward IAA and the rate of IAA transport, which in turn might play a crucial role in establishing the ideal auxin gradient for organogenesis in rice.

### PIN proteins are indispensable throughout the plant’s life cycle and are actively involved in growing and differentiating tissues

PIN proteins play a fundamental role in the plant’s growth and development. One of the first mutants identified in the auxin signaling pathway was *pin-formed1*, which produces naked “pin-like” stems without any flowers in *Arabidopsis*, and this typical phenotype later led to the PIN protein nomenclature (Weijers et al., 2018). Zhang et al. (2020b) explicitly demonstrated that the *cis*-regulatory and coding regions of PIN proteins of *Arabidopsis* coevolved and played a vital role in establishing dynamic auxin gradients across tissues leading to the evolution of the complex architecture of flowering plants. Furthermore, PINs are essentially found in multicellular plants ranging from early evolved green alga to latest evolved angiosperms that emphasize their critical role in the evolution of multicellular plants that require a complex spatiotemporal distribution of auxin. In rice, various PIN variants are found to be differentially expressed in all of the types of tissues including vegetative and reproductive organs (Wang et al., 2009; Miyashita et al., 2010). We also identified the PIN expression across various tissues and organs in rice. The famous *Avena* curvature test discovered the auxin-dependent phototrophic movement of *Avena sativa* coleoptile tips (Went, 1928; Went and Thimann, 1937; Went and White, 1939), thus highlighting the role of light in auxin signaling. Laxmi et al. (2008) found that light plays an essential role in the intracellular distribution of AtPIN2 in *Arabidopsis*. Expectedly, several light-responsive *cis*-regulatory elements were found in promoters of all of the rice PINs. The cross talk between auxin and other hormones determines the overall plant growth and development (Chandler, 2009; Munné-Bosch and Müller, 2013; Liu et al., 2014). We found auxin-, gibberellin-, cytokinin-, and ABA-responsive elements in promoters of rice PIN genes and identified phytohormone metabolism- and signaling-related genes that were co-expressed with rice PIN genes. These findings highlighted the indispensable involvement of PIN proteins throughout the plant’s life cycle.

The cellular differentiation process in plants strongly relies on the establishment of auxin gradients (Overvoorde et al., 2010). Furthermore, organogenesis requires a controlled production of new cells within meristematic tissues, and auxin is known to promote cell division, meristem maintenance, and establishment of cellular patterning (Perrot-Rechenmann, 2010). Since PINs are involved in auxin gradient formation, we assessed their expression pattern in non-expanding vs. expanding regions of leaf and shoots and their expression profile in organ-differentiating zones. The overall observations demonstrated that plants downregulate the PIN expression in non-expanding zones like root and shoot tips, while plants upregulate the PIN expression in elongating zones like leaf lamina (beyond leaf tips) and the root elongation zone (beyond leaf tips). Such regulation of PIN expression is critical to divert auxin from the tip regions where its concentration is the highest to growing zones where auxin can mediate both cell division and cell elongation. Furthermore, upregulation of the PIN expression in differentiating zones like root-stem junction and stem-leaf junctions indicates the importance of auxin flow in cellular differentiation and formation of newer organs.

### PIN genes respond to common abiotic stresses faced by rice plants: Heat, salinity, and drought

Global warming (or rising temperature), drought, and expansion in the area of saline soil are the major causes of concern for rice productivity worldwide. Heat stress affects plant growth and development by disrupting the stability of various proteins, membranes, and cytoskeleton structures. Drought stress decreases the relative water content in plants due to reduced soil water potential, whereas increasing soil salinity induces both osmotic and ionic stress in the plants (Korver et al., 2018). Since auxin is intricately involved in plant developmental processes and PIN transporters determine auxin flow, the PIN’s involvement in the modulation of abiotic stresses is natural. For instance, *OsPIN2* and *OsPIN5B* were found to be induced by drought, heat, and cold stresses (Du et al., 2013). The second alternate splice variant of *OsPIN3A* (there are three splice variants of *OsPIN3A*; **Table 1**), *OsPIN3t*, when overexpressed was found to confer drought tolerance in rice (Zhang et al., 2012). In wheat, Kumar et al. (2021) found multiple PIN genes to be upregulated during drought and heat stress. Xu et al. (2022) found induction of *OsPIN5B* and *OsPIN9* under various abiotic stress conditions. Our study revealed the upregulation of *PIN5C* and *PIN9* during salt stress and *PIN2*, *PIN5C*, and *PIN9* during drought stress. Thus, *PIN5C* and *PIN9* appear to be the ideal candidate PIN genes to develop multi-stress-tolerant transgenic rice. Our study indicated that during heat stress, the expression of several PIN members was reduced (*PIN1A*, *PIN1B*, *PIN3B*, *PIN5A*, and *PIN5B*), but the expression of other PIN members was unaffected. Kumar et al. (2021) found that most of the wheat PIN (*TaPIN*) family members are not involved in heat stress during the initial (1 h) and late (6 h) hours of treatment and found that *TaPIN37* is induced after 1 h of treatment and decreased thereafter at the sixth hour. Furthermore, *TaPIN14* and *TaPIN28* were upregulated during heat stress at the sixth hour of heat treatment. Since we treated the rice seedlings for 16 h and thereafter recorded PIN expression, it appears that a longer duration of heat treatment might downregulate PIN expression. Another study by Zhao et al. (2021) in pineapple (*Ananas comosus*) revealed that the expression of *AcPIN8* was upregulated under heat stress while *AcPIN5a* was downregulated. These observations indicate that the PIN expression is very dynamic, and they are differentially regulated in a PIN member- and plant species-dependent manner during heat stress.

### PIN and IAA biosynthesis genes are co-induced upon auxin treatment

Auxin biosynthesis and its PAT *via* PIN efflux transporters are interlinked phenomena. Auxin is typically synthesized in the shoot apical meristem and leaf promordia and transported to other plant tissues by polar transport machinery (Swarup and Péret, 2012). The next step in auxin biosynthesis involves its efflux from the cell and transport to neighboring ones *via* PIN. Therefore, we investigated whether the initiation of IAA biosynthesis and PIN expression are co-regulated, and we found that IAA is that molecular switch that not only induces its own synthesis but also induces the expression of PIN efflux transporters in the roots. PAT is important for adventitious root emergence and growth in rice (Lin and Sauter, 2019). Co-induction of both these categories of genes in roots of rice indicates that cells become prepared for IAA transportation once it is synthesized in the source regions.

### PIN genes are involved in shaping an auxin-dependent root architecture in rice

**Figure 11:**
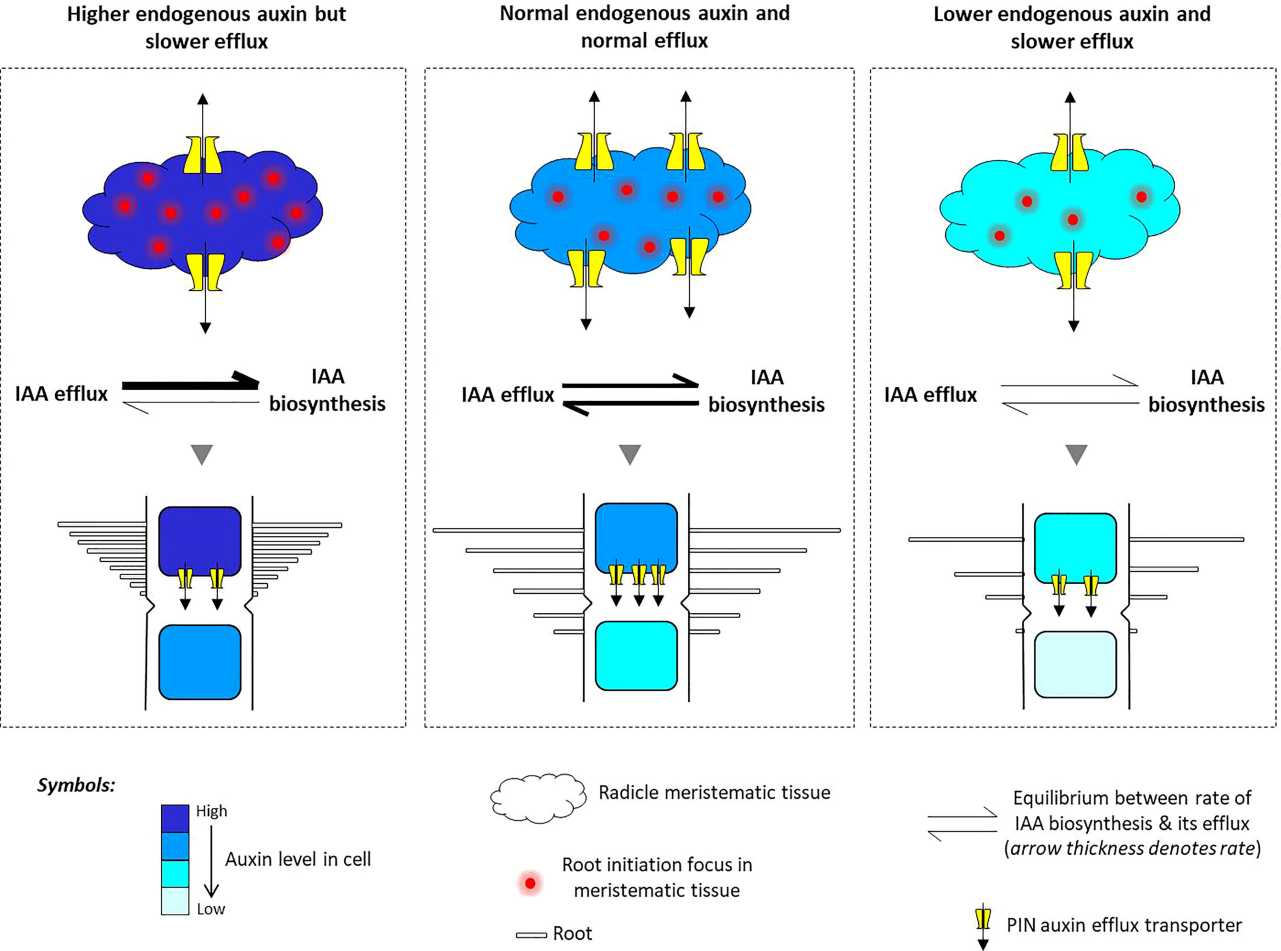
A model of PIN-mediated determination of auxin flow and its role in shaping an auxin-dependent root architecture in rice. The meristematic cells present in the radicle of rice embryo undergo normal cell division to put forth the usual root architecture in rice seedlings. But upon experiencing high auxin inflow and greater auxin biosynthesis, the meristematic cells start to rapidly divide and initiate more foci of root initiation, as excess auxin cannot be quickly flushed out due to the downregulation of the PIN auxin efflux transporters. This results in the formation of excessive secondary roots from multiple foci of root initiation. On the contrary, when the cellular auxin level decreases by a lower IAA biosynthesis and auxin flow is inhibited by TIBA treatment, PIN downregulation causes a restriction in the movement of endogenous auxin, resulting in lesser meristematic cell division, lesser number of root initiation foci, and lesser secondary root formation. Overall, there exists a fine balance between the rate of auxin biosynthesis and their PIN-mediated efflux. When in equilibrium, there is normal root production, but when equilibrium shifts toward high endogenous auxin, it favors excessive cell division and differentiation into a higher number of roots. Furthermore, the combined influence of lower auxin biosynthesis and lesser auxin transportation decreases the number of roots.

Although a brief duration of external auxin treatments could induce PIN expression in roots, we found that a longer duration of auxin treatments led to the downregulation of PIN expression in the roots. However, under such condition, the expression of the IAA biosynthesis genes either remains unchanged or is upregulated. Thus, it appears that beyond the threshold limit, IAA functions as a negative regulator switch for PIN expression but acts as a positive regulator switch for IAA biosynthesis. Such differential regulation of both categories of genes has an important bearing on rice root development and architecture. Auxin treatment was found to promote shorter but higher numbers of roots in rice seedlings. Yamamoto et al. (2007) showed that overexpression of the *OsYUCCA1* gene in rice resulted in profuse root growing in random directions. We also witnessed the same when plants were externally supplied with auxin. This indicates that the cellular auxin content is directly proportional to the number of root formation. A very interesting and detailed investigation by Benková et al. (2003) showed that in *Arabidopsis*, 2,4-D (an auxin) treatment disrupts the formation of the auxin gradient in the developing root primordia wherein auxin was shown to be uniformly distributed over the entire root primordia unlike the tip region, which happens under normal circumstances. Based on these information and data, we propose a model (**Figure 11**) where we suggest that when meristematic cells of the radicle experience external auxin stimuli, the endogenous auxin level of these cells increases either by direct uptake or by increased biosynthesis of auxin, which in turn disrupts the formation of the polar auxin gradient by downregulation of PIN efflux transporters. These activities in the root primordia lead to apolar localization of auxin in the entire root primordia, and since auxin is actively involved in cell division, more foci of root initiation appear in the root primordia, leading to the formation of a higher number of secondary roots in rice.

We observed that TIBA treatments led to the downregulation of PIN and auxin biosynthesis genes in roots, and there was a reduction in the number of roots formed. TIBA is a PAT inhibitor (Teale and Palme, 2018). We indeed found that TIBA hampered PAT in the roots of rice seedlings (**Figure 8**). Furthermore, since TIBA treatments were found to downregulate the expression of auxin biosynthesis genes, it might be possible that the TIBA-mediated PAT disruption does not directly bring about downregulation of PIN genes, rather, lower biosynthesis of auxin in the roots facilitates PIN downregulation. Here, we use the same model (**Figure 11**) to propose that inhibition of PAT not only affects auxin transportation but also favors low auxin biosynthesis in the meristematic cells of the radicle. Benková et al. (2003) showed that similar to 2,4-D treatment, the NPA (N-1-naphthylphthalamic acid) (an auxin transport inhibitor having a similar action as TIBA; Teale and Palme, 2018)-mediated disruption of PAT impairs the establishment of the auxin gradient in the root primordia of *Arabidopsis*. In case of rice, a low auxin level in the root primordia, a loss of auxin gradient formation, and a lower rate of auxin translocation favor lesser numbers of foci for secondary root initiation and therefore lesser root formation. Xu et al. (2005) showed that in RNAi lines of *OsPIN1A*, TIBA treatment resulted in a lesser number of root formation, which again indicates that the combined influence of TIBA and PIN downregulation has an inhibitory effect on root formation in rice seedlings. Overall, it appears that there exists a fine balance between the rate of cellular auxin biosynthesis and its efflux by auxin transporters. When in balance, it favors normal cell division and root formation. But when the balance shifts toward more cellular auxin content, excessive cell division and multiple root formation take place. Furthermore, a slower rate of auxin synthesis and its slower rate of transportation result in lesser root formation (**Figure 11**).

## Conclusion

In this study, we analyzed 12 PIN genes distributed over eight chromosomes of rice. All of the PIN proteins have typical well-conserved 10 TM domains with one variable HL domain containing potential MAPK phosphorylation sites. Seven of the PIN members possess long HL domains having potential phosphorylation sites. Typical of PIN members, rice PINs were predicted to have the usual cell membrane localization. Additionally, PIN3 and some of the short HL domain-containing PINs (PIN1D and PIN8) had organellar localization predictions. The spatiotemporal expression analysis highlighted the dynamic expression pattern of PIN members. Usually, the PIN expression level is higher in growing and differentiating regions of rice. The PIN genes are also auxin-responsive particularly in the root tissues. Additionally, we found that *PIN5C* and *PIN9* are the potential common candidates for developing salt- and drought-tolerant rice. Interestingly, we found that the cellular auxin level functions as a molecular switch to differentially turn on and off the expression of PIN and IAA biosynthesis genes, which results in an auxin-dependent variable root architecture in rice. Our study paves the way for developing stress-tolerant rice and plants with a desirable root architecture by genetic engineering.

## Data availability statement

The original contributions presented in the study are included in the article/**Supplementary Material**. Further inquiries can be directed to the corresponding author.

## Author contributions

AS conceived the research plans and supervised the experiments. MM performed all the bioinformatics analyses and experiments. RB performed photography and constructed virtual root images. NA helped in data analysis. MM wrote the manuscript which was proof read by AS. All authors contributed to the article and approved the submitted version.

## Funding

MM gratefully acknowledges National Post-Doctoral Fellowship (NPDF; File number: PDF/2020/000511) Award from Science and Engineering Research Board (SERB), Government of India. AS acknowledges Sir J.C. Bose National Fellowship Award from SERB, Government of India.

## Acknowledgments

The authors sincerely thank Dr. Jitender Giri, NIPGR, New Delhi, India for kindly providing the DR5-*Gus* rice seeds.

## Conflict of interest

The authors declare that the research was conducted in the absence of any commercial or financial relationships that could be construed as a potential conflict of interest.

## Publisher’s note

All claims expressed in this article are solely those of the authors and do not necessarily represent those of their affiliated organizations, or those of the publisher, the editors and the reviewers. Any product that may be evaluated in this article, or claim that may be made by its manufacturer, is not guaranteed or endorsed by the publisher.
