# Supplementary material for: Characterization and expression profiling of PIN auxin efflux transporters reveal their role in developmental and abiotic stress conditions in rice
Source: Front Plant Sci. 2022 Dec 1;13:1059559. doi: 10.3389/fpls.2022.1059559 (PMC9751476; doi:10.3389/fpls.2022.1059559)
Supplement: Supplementary file 1 [file DataSheet_1.pdf]

SUPPLEMENTARY FILE

Characterization and expression profiling of PIN auxin efflux transporters reveals role in developmental and abiotic stress conditions in rice

Mrinalini Manna[a], Balakrishnan Rengasamy[a], Navin Kumar Ambasht[b], Alok Krishna Sinha[a,*]

[a]National Institute of Plant Genome Research, Aruna Asaf Ali Marg, New Delhi – 110067, India

[b]Department of Botany, Christ Church College, Kanpur – 208001, India

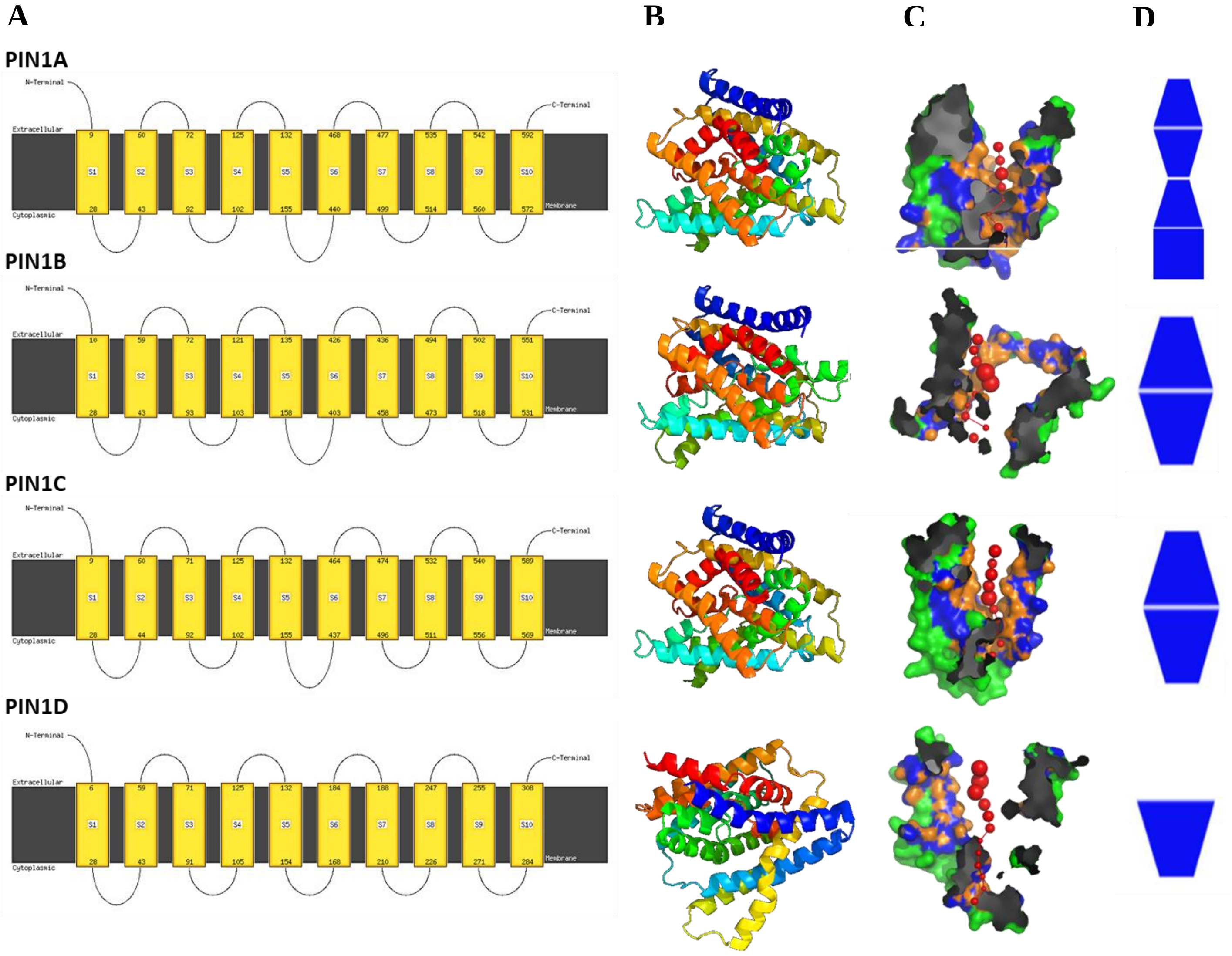

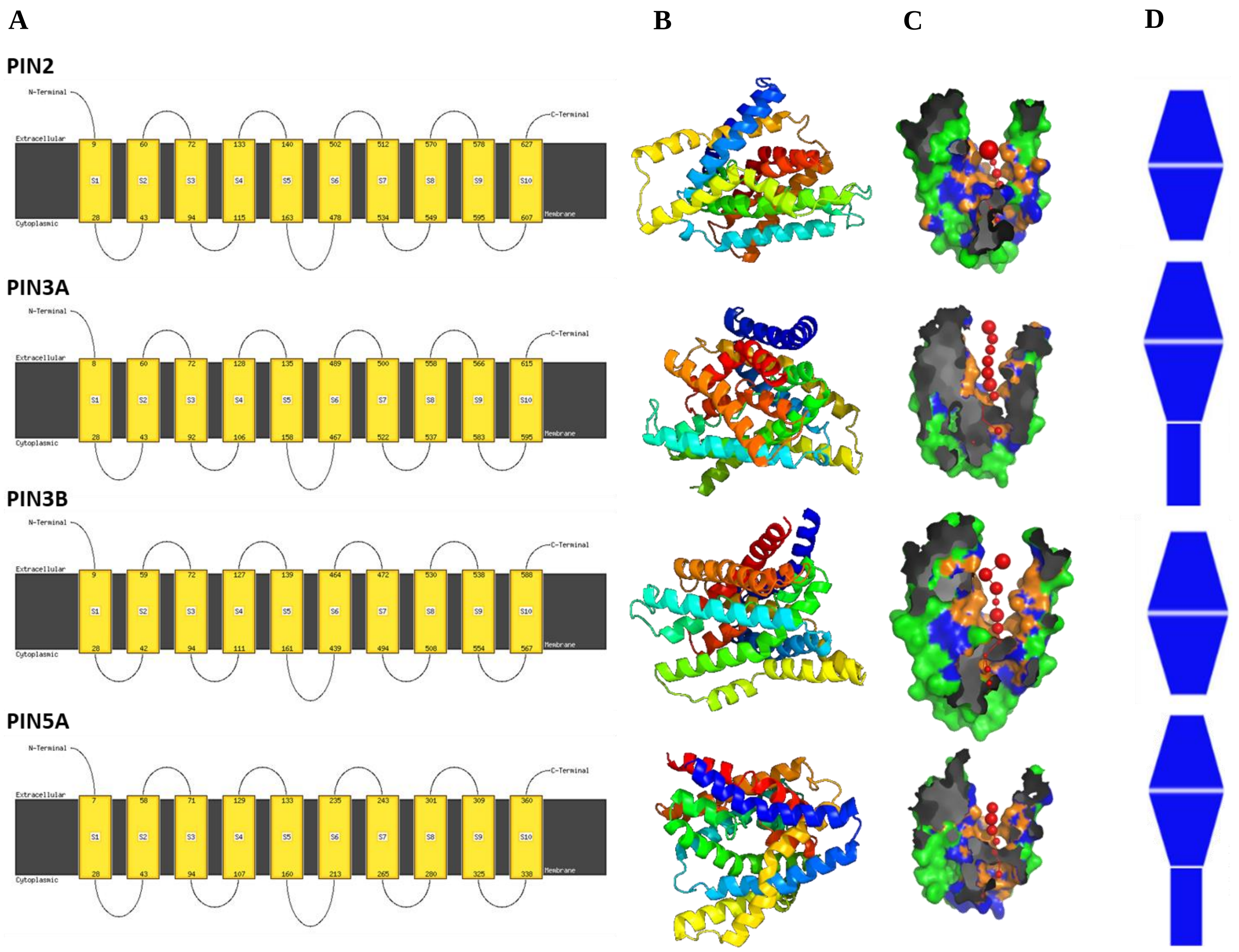
A
B
C
D
PIN2
PIN3A
PIN3B
PIN5A
N-Terminal
C-Terminal
Extracellular
Cytoplasmic
Membrane
S1
S2
S3
S4
S5
S6
S7
S8
S9
S10

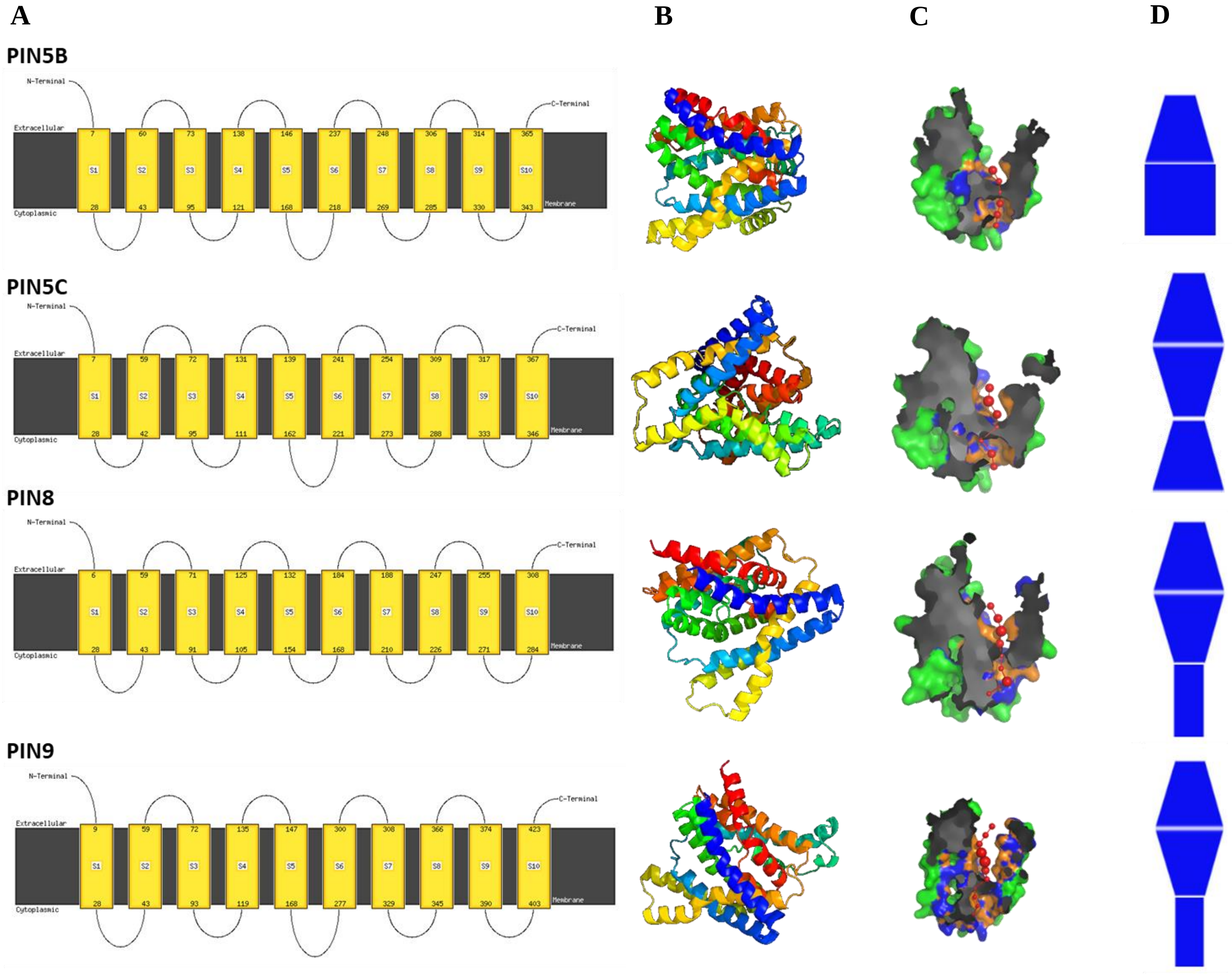

Supplementary figure 1 Predicted three-dimensional (3D) models of 12 rice PIN proteins. **(A)** Cell membrane organization of PIN proteins indicate 10 transmembrane domains and one larger hydrophilic loop (HL) domain present between HL domain 5 and 6. **(B)** Predicted 3D protein structure of PIN proteins indicates presence of 10 α helices which constitute 10 transmembrane domains. **(C)** Pore morphology of the PIN proteins. **(D)** Pore shapes of PIN proteins indicate varying pore dimensions and morphologies.

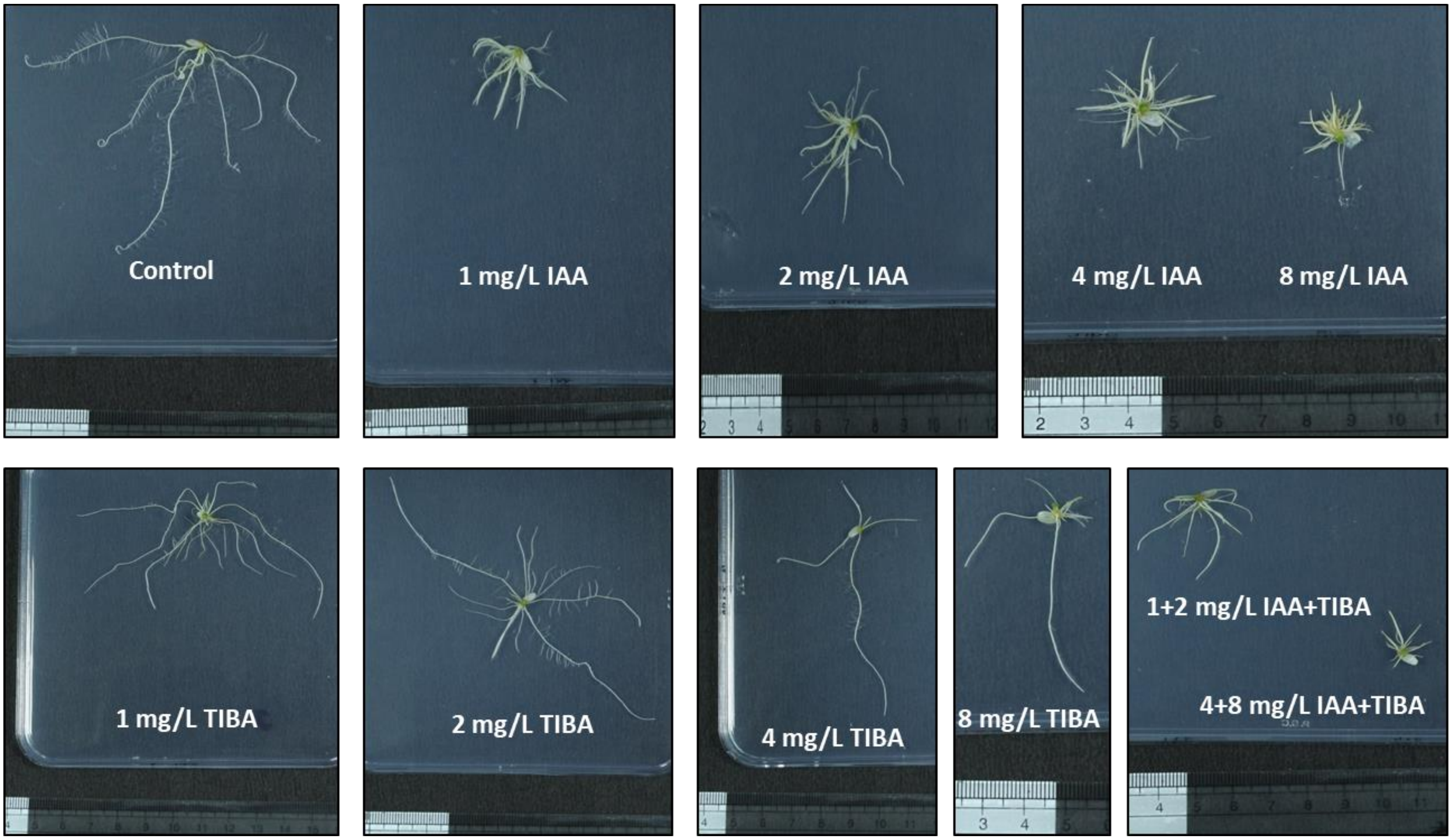

Supplementary figure 2 Actual root images of rice germinated and grown over media containing various concentrations of IAA and TIBA. Control denotes no external supplementation of IAA or TIBA. The virtual root images were made based on these images.

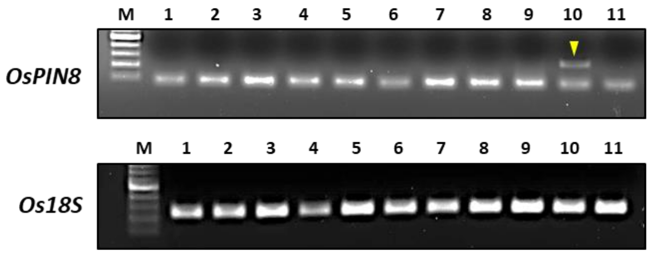

M: 100 bp ladder
1: control shoot
2: control root
3: shoot from 3h IAA treated seedlings
4: root from 3h IAA treated seedlings
5: control seedling
6: heat treated seedling
7: salt treated seedling
8: drought treated seedling
9: mature panicle
10: upper half of immature panicle
11: lower half of immature panicle

Supplementary figure 3 Analysis of *OsPIN8* gene expression in various rice tissue/ treatment samples by semi-quantitative RT-PCR (with 35 PCR cycles). Out of 11 samples tested, *PIN8* expression (299 bp) was only evident in upper half of rice immature panicle (indicated with yellow arrow). The lower bands below 100 bp ladder position in *OsPIN8* RT-PCR lane are the primer dimers. *Os18S* gene specific RT-PCR (236 bp) was done to analyze quality of cDNA and depict equivalent cDNA template used in RT-PCR.

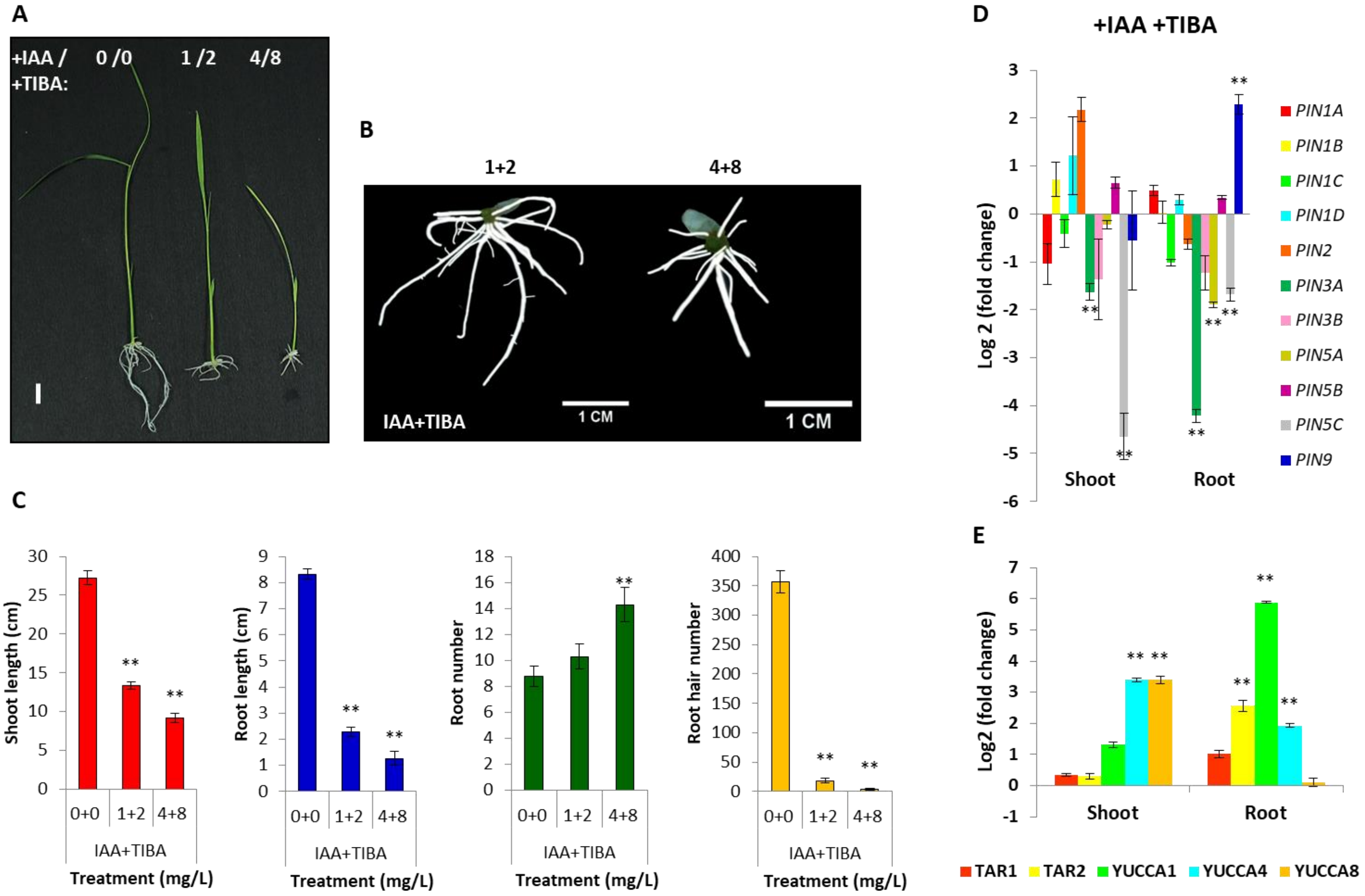

Supplementary figure 4 Seeding and root morphologies and expression profiling of PIN and IAA biosynthesis genes in shoot and root tissues of rice seedlings grown over half MS media containing 1 mg/L IAA + 2 mg/L TIBA or 4 mg/L IAA + 8 mg/L TIBA for a duration of 15 days. **(A)** Seedling morphologies at respective treatments. **(B)** Magnified root images under respective conditions. **(C)** Quantification of shoot lengths upon IAA + TIBA treatment revealing reduction in shoot lengths. There was reduction in root lengths as well. The 4 mg/L IAA + 8 mg/L TIBA treatment significantly increased the root numbers and there was drastic reduction in rot hair formation. **(D)** Expression pattern of PIN genes in 4 mg/L IAA + 8 mg/L TIBA treated rice seedlings (n=3) as measured by qRT-PCR. PIN3A was significantly downregulated in shoots. While, PIN3A, 5A and 5C were significantly downregulated in root tissues, PIN9 was significantly upregulated in root. **(E)** Expression profiling of IAA biosynthesis genes in IAA treated rice seedlings (n=3) showing upregulation of YUCCA4 and 8 in shoot and TAR2, YUCCA1 and 2 in root.

Supplementary table 1: Primers used in the study (all the primers belong to respective rice genes)

Gene name	Forward primer (5'→3')	Reverse primer (5'→3')
PIN1A	CGTCTGCTTCAGGTGGAACT	GGTGATAGGCAAGGCGATGA
PIN1B	TGGTCCCTCGTCTCCTACAG	GCAAACACAAAGGGCACGAT
PIN1C	CTCATCGGCCTCATCTGGTC	AGCCCCAGCAGGATGTAGTA
PIN1D	AGGTGGGGAATTGAGATGCC	GCCATGGCATACGAAGCAAG
PIN2	GCGCAAGCTCATCAGAAACC	GCAAATGTCGCAACGGTCTT
PIN3A	GGCCATGTTTAGCCTGGGAT	TTACCGCTGTGCTCAGGATG
PIN3B	CCATTCTCTCCGATGCAGGG	GGCACAATTCCTTGTGGCAG
PIN5A	ATGTCCAAGTCAGGCACAGG	AGCATGCAGCCCGTATTCTT
PIN5B	GGGTTTGTTCATGGCGTTGC	GAAGCGCAGCCTGTATGATG
PIN5C	GGGCTTCATGCCGATGTACT	TAGACAAAGCCCAGAACCGC
PIN9	GTCATCTGGATGGCGGTGAA	AATGATGTCACTGCCAGGGG
18S	TTAGGCCACGGAAGTTTGAG	GTACAAAGGGCAGGGACGTA
TAR1	GAAGGAAGGGGTGGACGAC	AGTTCATGGCGGCGAGG
TAR2	GGTGCGATAGGGAGGATGTG	CGAGAGGCGGTTGATGAAGA
YUCCA1	TTGGGACGCTAGACCACATC	CAAGTCACCGGCATCCTTGA
YUCCA4	ATGGCGTGGAGTTTGTGGAT	GCCTTGAGAAACCAACAGCG
YUCCA8	TGGTCTCAAGAGGCCCAAAC	TCCGTGAACAACTCACCGTC
PIN8	TCCTACAAAAGTCGCTTGCC	CAGTGCCCAAATTAAACCTGTAGT

Supplementary table 2: Genes names, IDs, putative functions and their classification w.r.t. Fig. 5

**Node names in Fig. 5**	**Full Name**	**Uniprot ID**	**Function of the protein**	**Category**
aa transporter	Amino acid transporter family protein	Q53MQ4	Amino acid transportation	Amino acid transporters
aa transporter3	Auxin transporter-like protein 3	Q7XGU4		
aa carrier	Amino acid transporter family protein	Q53LH2		
K-transporter	High affinity potassium transporter	Q7XIV8	A membrane associated protein associated with potassium transportation	Potassium transporter
ABC transporter	ABC transporter	Q0J9M8	In a cell, ABC transporters can facilitate both influx and efflux of auxin	IAA transporters
ABC	ABC transporter	Q6YUU5		
ABC19	ABC transporter B family member 19	A0A0P0WBD9		
ABCG32	ABC transporter G family member 32	Q8LQX2		
ABCG43	ABC transporter G family member 43	Q6GU86		
AUX1	AUX1	Q10P71	AUX1 protein, transmembrane localized, aids in auxin import inside cell	
Auxin transporter-2	Auxin transporter-like protein 2	Q688J2	Proton driven auxin influx transportation	
Auxin transporter-4	Auxin transporter-like protein 4	Q53JG7		
LAX-1	LAX PANICLE 1	Q7XAQ6	Auxin transporter-like protein 1, Involved in organogenesis, regulation of shoot branching by controlling axillary meristem formation	
PIN like 2	PIN like protein	A0A0P0XD33	A putative PIN like auxin efflux transporter	
PIN1A	Auxin efflux carrier 1A	Q5SMQ9	Effluxes auxin out of the cell and facilitates polar	
PIN1B	Auxin efflux carrier 1B	P0C0X5		
PIN2	Auxin efflux carrier 2	Q651V6		
PIN3A	Auxin efflux carrier 3A	Q5VP70		
PIN3B	Auxin efflux carrier 3B	Q6L5F6		
PIN5A	Auxin efflux carrier 5A	Q5JLM1		
PIN5C	Auxin efflux carrier 5C	Q6ZIB5		
PIN9	Auxin efflux carrier 9	Q5VQY3		
PID-1	Protein kinase PINOID	Q6ASU0	Phosphorylates hydrophilic loop domains of PIN auxin efflux carriers to facilitate their polar localization in cell membrane	Regulator of polar localization of PIN proteins in cell membrane
PID-2	Protein kinase PINOID	Q2QM77		
FC monooxygenase-1	Flavin-containing monooxygenase	Q7XP26	A flavin containing monooxygenase, involved in auxin biosynthesis	IAA biosynthesis and Metabolism
FC monooxygenase-2	Flavin-containing monooxygenase	Q7XHL8		
IAA synthatase	Probable indole-3-acetic acid-amido synthetase GH3.7	Q654M1	Associated with IAA biosynthesis	
TAR 1 like	Tryptophan aminotransferase-related protein 1	Q0DKE8	Putatively involved in auxin biosynthesis	
TAR like-2	Tryptophan aminotransferase-	Q5VQG8		

	related protein 2			
ILR1-like 5	IAA amino acid hydrolase	Q7XUA8	Hydrolyzes certain amino acid conjugates of IAA	
Nexin 1	Putative sorting nexin-1	Q5N7G9	Protein sorting nexin 1 class of proteins are involved in auxin homeostasis	
IAA31	Auxin-responsive protein IAA31	P0C133	Auxin-responsive protein IAA31, Aux/IAA proteins are short-lived transcriptional factors that function as repressors of early auxin response genes at low auxin concentrations	Negative regulator of auxin responsiveness
SAUP15A	Auxin induced protein 15A	Q0J8D3	A probable SAUR gene whose RNA functions as a negative regulator of auxin synthesis, transport and organ growth	
SAUR32	Small auxin upregulated RNA 32	Q5Z7T1	Functions as a negative regulator of auxin synthesis, transport and organ growth	
SAUR36	Small auxin upregulated RNA 36	Q6H664		
SAUR36	Small auxin upregulated RNA 36	B7F8P5		
SAUR76	Auxin-responsive protein SAUR	Q6ZKQ7		
ARF1	Auxin Response Factor 1	Q5NB85	Transcription factor that binds to 5'-TGTCTC-3' found in auxin responsive promoter elements	Positive regulator of auxin responsiveness
ARF11	Auxin response factor 11	Q8S983		
EIP	Ethylene Insensitive 3-like 5 protein	A0A0P0VKR0	An ethylene insensitive (EIN) class of transcription factor, activated by MAP kinases, required for ethylene responsiveness in plants	Ethylene responsiveness
ACC oxidase	1-aminocyclopropane-1-carboxylic acid (ACC) oxidase	A0A0P0V4I5	Putative 1-aminocyclopropane-1-carboxylic acid(ACC) oxidase, involved in ethylene biosynthesis	Ethylene biosynthesis
GR factor 11	Growth-regulating factor 11	Q6AWX8	TF that plays an active role in gibberellin induced stem elongation	Gibberellin responsiveness
bHLH-106	Basic helix-loop helix domain containing TF-106	Q6AT90	Binds with TF ILI6 to form a heterodimer and regulates grain length and weight by controlling cell elongation in lemma and palea	Overall plant growth and development
Hydroxysteroid dehydrogenase	Hydroxysteroid dehydrogenase	Q2R3W3	Associated with regulation of plant growth and development and likely promotes brassinosteroid signaling in plants	
LPA1	P-loop NTPase domain-containing protein LPA1	B9F4I8	Required for the accumulation of phytic acid in seeds	
LTAIC	Leaf and tiller angle increased controller	Q5Z807	Mediates optimum plant angle architecture through brassinosteroid (BR) signalling. May act as negative regulator in sterol homeostasis and negative regulator of BR signalling. Binds to BZR1 promoter to negatively regulate it and attenuate BR signalling to regulate leaf bending.	
Neutral invertase	Alkaline/ neutral invertase	Q5ZA22	Associated with overall plant development	
O-fucosyltransferase	O-fucosyltransferase family protein	A0A0P0WDP9	Associated with cell wall biosynthesis	
p-glycoprotein 1	Putative p-glycoprotein 1	Q7EZL2	Associated with long distance auxin transportation and overall plant development	
RAC4	Rac-like GTP binding	Q67VP4	Complexes with Rho-GDP-dissociation	

	protein		inhibitors (Rho GDIs). Released from GDI protein in order to in order to translocate it to membranes upon activation	
RAC5	Rac-like GTP binding protein	Q6EP31	Complexes with Rho-GDP-dissociation inhibitors (Rho GDIs). Released from GDI protein in order to in order to translocate it to membranes upon activation	
S-formylglutathione hydrolase	S-formylglutathione hydrolase	Q5JLP6	A serine hydrolase involved in detoxification of formaldehyde	
Shikimate kinase like 2	Shikimate kinase like 2	Q336N7	A probable inactive shikimate kinase like 2 protein (chloroplast localized)	
SHORT-ROOT 1	Protein SHORT-ROOT 1	Q8H2X8	TF required for asymmetric cell division involved in radial pattern formation in roots	
UDP-N-Ac-DPNAcPT	UDP-N-acetylglucosamine phosphotransferase	Q7EZZ0	Associated with protein glycosylation	
WUS	WUSCHEL-related homeobox 1	Q7XM13	WUSCHEL-related homeobox 1A, transcription factor associated with developmental processes	
PP2A	Phosphatase 2A regulatory subunit	Q6K4K9	Protein phosphatase 2A characterized to have a positive role in providing sheath blight resistance to rice	Rice sheath blight resistance
A0A0N7KKZ6	Uncharacterized proteins	A0A0N7KKZ6	Unknown	Unclassified
Q6F389		Q6F389		
Q6Z697		Q6Z697		
Q7XUC8		Q7XUC8		

Supplementary table 3: Rice seedling growth parameters under influence of IAA and TIBA

Treatment	Specific treatment conditions	Shoot length (cm)	Root length (cm)	Root number	Root hair number
Control	No externally applied IAA/ TIBA	27.24±0.92^{a}	8.33±0.20^{a}	8.8±0.79^{a}	356.8±18.65^{a}
IAA	1 mg/L IAA	17.16±0.61^{ab}	3.63±0.33^{ab}	11.2±0.79^{a}	52.4±7.16^{ab}
	2 mg/L IAA	16.04±0.65^{a}	2.09±0.21^{a}	14.1±0.87^{a}	29.2±4.82^{a}
	4 mg/L IAA	15.49±0.43^{ac}	1.42±0.15^{a}	17.3±0.82^{ab}	20.2±2.86^{ac}
	8 mg/L IAA	12.47±0.58^{a}	1.13±0.13^{a}	25.5±2.07^{a}	18.4±1.82^{a}
TIBA	1 mg/L TIBA	18.73±0.90^{a}	7.96±0.21^{ns}	10.3±0.82^{a}	93.8±7.63^{a}
	2 mg/L TIBA	14.58±1.49^{a}	7.19±0.27^{a}	7.4±0.52^{ns}	89.8±8.96^{a}
	4 mg/L TIBA	13.99±1.55^{a}	6.92±0.26^{a}	6.3±0.48^{a}	37.6±7.40^{a}
	8 mg/L TIBA	10.52±0.94^{a}	5.50±0.19^{a}	5.8±0.79^{a}	10.6±2.41^{a}
IAA+TIBA	1 mg/L IAA + 2 mg/L TIBA	13.33±0.45^{ab}	2.27±0.17^{ab}	10.3±0.95^{ns}	18.4±3.85^{ab}
	4 mg/L IAA + 8 mg/L TIBA	9.17±0.63^{ac}	1.26±0.26^{a}	14.3±1.34^{ab}	4.2±1.48^{ac}
